# Bismuth Subnitrate-Catalyzed Markovnikov-Type Alkyne Hydrations under Batch and Continuous Flow Conditions

**DOI:** 10.3390/molecules26102864

**Published:** 2021-05-12

**Authors:** Zsanett Szécsényi, Ferenc Fülöp, Sándor B. Ötvös

**Affiliations:** 1 Institute of Pharmaceutical Chemistry, University of Szeged, Interdisciplinary Excellence Center, Eötvös u. 6, H-6720 Szeged, Hungary; zsanettszecsenyi@gmail.com; 2 MTA-SZTE Stereochemistry Research Group, Hungarian Academy of Sciences, Interdisciplinary Excellence Center, Eötvös u. 6, H-6720 Szeged, Hungary; 3 Institute of Chemistry, University of Graz, NAWI Graz, Heinrichstrasse 28, A-8010 Graz, Austria

**Keywords:** alkynes, bismuth subnitrate, continuous flow, alkyne hydrations, methyl ketones

## Abstract

Bismuth subnitrate is reported herein as a simple and efficient catalyst for the atom-economical synthesis of methyl ketones via Markovnikov-type alkyne hydration. Besides an effective batch process under reasonably mild conditions, a chemically intensified continuous flow protocol was also developed in a packed-bed system. The applicability of the methodologies was demonstrated through hydration of a diverse set of terminal acetylenes. By simply switching the reaction medium from methanol to methanol-*d*_4_, valuable trideuteromethyl ketones were also prepared. Due to the ready availability and nontoxicity of the heterogeneous catalyst, which eliminated the need for any special additives and/or harmful reagents, the presented processes display significant advances in terms of practicality and sustainability.

## 1. Introduction

The pursuit of environmental protection and the implementation of sustainability have become an important endeavour in current organic chemistry [1,2,3]. However, in many cases, synthetic protocols utilize catalytic materials that rely on rare, expensive, toxic or harmful metal components [4]. Truly sustainable and economically reliable synthetic methodologies must be free of such elements and should ideally rely on cost-efficient, nontoxic and readily available catalysts [5]. In this manner, bismuth and its compounds are of significant current interest [6,7]. This is mainly due to the fact that, in contrast to other heavy metals, bismuth and its compounds are neither toxic nor harmful for the environment, while being stable to air, easy to handle and inexpensive [8,9,10]. Importantly, bismuth(III) compounds are known to exhibit remarkable Lewis acid character, and they are capable of activating diverse substrates, such as alcohols, amines, olefins and alkynes [11,12,13,14,15]. Recent examples for the catalytic utility of bismuth(III) compounds include oxidations, deprotections, acetal formations and, esterifications, as well as various carbon–carbon bond formations and heterocyclizations [16,17,18,19,20,21,22,23,24]. Catalytic sources for such reactions are generally soluble salts, such as Bi(OTf)_3_, BiBr_3_, BiCl_3_ and Bi(NO_3_)_3_·5H_2_O [16], whereas heterogeneous bismuth(III) catalysts are extremely scarce [25]. Although most of the typical bismuth(III) salts are relatively cheap, the application of heterogeneous sources of the catalytic metal would involve obvious benefits, from environmental and practical aspects (e.g., facile product isolation and catalyst reusability).

The Markovnikov-type hydration of alkynes is a well-known process yielding valuable carbonyl compounds [26,27]. Its synthetic utility can be explained by the ease of introduction of the acetylene moiety, which can practically be regarded as a carbonyl equivalent upon unmasking by hydration [28]. Traditional alkyne hydrations employ catalytic amounts of mercuric salts in strongly acidic media, which involves obvious environmental concerns [27]. Recently, numerous transition metal salts and complexes were found applicable as catalysts for Markovnikov-type alkyne hydration—among these, gold, silver, platinum, palladium, rhodium and ruthenium compounds [29,30,31,32,33,34,35,36,37,38]. Although, these represent great progress compared to the classical methodologies, such reactions are generally promoted by soluble catalytic sources typically in the presence of various ligands. This fact, along with the high price of the catalytic metal, should be considered as a significant drawback.

Bismuth subnitrate (Bi_5_O(OH)_9_(NO_3_)_4_) is a commercially available bismuth(III) compound that bears significant medical uses (e.g., as an antidiarrheic agent) [39]. It can readily be prepared by the controlled thermal decomposition of Bi(NO_3_)_3_·5H_2_O [40], and it is practically insoluble in most typical organic solvents. In spite of its beneficial properties, such as ready availability, nontoxicity and low price, it has scarcely been investigated as a heterogeneous bismuth catalyst in organic synthesis [41,42]. Earlier, we reported that various soluble bismuth salts are useful as homogeneous catalysts in alkyne hydration [20]. We speculated that bismuth subnitrate may be applicable as a heterogeneous source for catalytic bismuth(III) and that it may prove useful as an efficient heterogeneous catalyst for Markovnikov-type alkyne hydrations. The application of heterogeneous catalysts in continuous flow systems have received an upsurge of interest, which is due to numerous benefits, such as facile catalyst handling, recycling and reuse, as well as simple product isolation [43,44,45,46,47,48,49]. Additionally, in loaded catalyst columns, continuous substrate streams interact with a superstoichiometric amount of catalyst species, which enhances the reaction rates considerably [50,51], while the improved control over temperature and residence time ensures a high selectivity and low waste generation [52,53,54,55]. We therefore intended to study the reactions not only under the traditional batch conditions but, also, within a continuous flow packed-bed reactor environment. Our results are presented herein.

## 2. Results and Discussion

As the starting point of our study, the catalytic activity of different bismuth(III) compounds was compared using the Markovnikov-type hydration of *p*-methoxyphenylacetylene as the model reaction (Table 1). The reaction mixture containing the alkyne (1.0 M), together with 15 mol% of the selected catalyst, was refluxed for 24 h in MeOH as the solvent. Having confirmed the lack of conversion without any catalyst present (entry 1), we were delighted to find that, in the presence of bismuth subnitrate as the catalyst, the corresponding methyl ketone was formed in a quantitative and selective reaction (entry 2). Bi(OTf)_3_ also furnished the quantitative conversion; however, dimethyl acetal **2** was detected as a side product to an extent of 12% (entry 3). According to the reaction mechanism suggested earlier [56,57], **2** is, in fact, an intermediary product, which is formed by the hydroalkoxylation of the alkyne with methanol; **2** is then hydrolyzed to yield methyl ketone **1** as the desired product. BiBr_3_ proved less reactive as a catalyst and furnished only 45% conversion, along with some notable amount (8%) of **2** formed as a side product (entry 4). Bi(OAc)_3_ and Bi_2_O_3_ proved inactive as a catalyst in the model reaction (entries 5 and 6).

Next, the effects of the most important reaction conditions were investigated carefully. As concerns the reaction time, it was found that 24 h is necessary for completion of the model reaction under reflux conditions in MeOH (further conditions: 15 mol% catalyst loading and 1 M substrate concentration). Shorter reaction times gave lower conversions—for example, 73% in the case of 12 h and 18% in the case of 3 h (Table 2, entries 1–5). Upon investigating the effects of catalyst loading (entries 6–8), the best results were achieved with 15 mol%; however, with only 2 mol% of bismuth subnitrate catalyst present, an acceptable conversion of 62% could still be achieved. Importantly, in the cases of 2 and 5 mol% catalyst loading, dimethyl acetal **2** appeared as a side product to an extent of 29% and 12%, respectively. Heating at reflux temperature was found to be necessary for efficient alkyne hydration, since only traces of product formation occurred at room temperature (entry 9). Upon increasing the substrate concentration to 2 M, a notable decrease of the conversion and selectivity occurred (entry 10), and 1 M was therefore considered as an optimum value later on.

Besides MeOH, the model reaction was attempted using H_2_O and different alcohols as the reaction medium (Table 3). In EtOH and *i*PrOH, a drastic reactivity decrease occurred that may be explained by the increased steric hindrance compared with MeOH during the hydroalkoxylation of the alkyne (entries 1–3) [57]. The hydration process furnished methyl ketone **1** in H_2_O also, but in this case, the conversion decreased to 69% (entry 4). In dry MeOH as the solvent, dimethyl acetal **2** was detected in the product solution to an extent of 8% (entry 5). This corroborates that trace amounts of water are necessary for effective hydrolysis of the dimethyl acetal intermediate into the desired methyl ketone product. Water traces may come from the moisture present in the air, from the solvent applied and/or from the catalyst itself. Additionally, it has been discussed earlier that bismuth(III) may bear some catalytic activity in the hydrolysis of the acetal intermediate [20], thus explaining the appearance of **2** upon decreasing the catalyst loading to 2 or 5 mol% (Table 2, entries 7 and 8).

After acquiring sufficient data on the effects of the reaction conditions, the reactivity of various alkynes was next explored. Amongst aromatic alkynes, phenylacetylene and its *p*-methyl-, *p*-ethyl and *p*-methoxy-substituted derivatives, as well as multi-methyl/methoxy-substituted phenylacetylenes, exhibited excellent reactivities with conversions in the range of 60–100% (Table 4, entries 1–4, 6 and 7). Furthermore, 3-ethynylthiophene, ethynylferrocene and ethyl propiolate also proved as good substrates and gave conversions in the range of 74–100% (entries 8–10). *p*-(tert-butyl)phenylacetylene gave only a moderate conversion, which is presumably due to steric effects (entry 5). In all the reactions investigated, the desired methyl ketones were formed selectively. Note that isolated yields were also determined in some representative instances.

Deuterium-labeled compounds have numerous applications—for example, as analytical standards or for the evaluation of the metabolic pathways or in tracer studies to investigate pharmacokinetics, catalytic cycles and reaction pathways [58,59,60]. The incorporation of deuterium into various organic compounds has therefore gained significant importance in medicinal, analytical and pharmaceutical chemistry [61]. In most cases, deuteration methods involve halogen/D exchange and are typically mediated by strong bases, which severely limits the functional group tolerance [62]. Moreover, catalytic H/D exchange reactions are also known; however, these often involve selectivity and/or environmental issues [63,64,65]. Inspired by these limitations, we explored a facile methodology to prepare valuable trideuteromethyl ketones by simply switching the reaction medium from MeOH to MeOH-*d*_4_ in bismuth subnitrate-catalyzed alkyne hydrations. As shown in Table 5, high conversion and 100% chemoselectivity were achieved in all the reactions investigated, and deuteration was highly favored over incidental hydrogen incorporation, as demonstrated by deuterium contents of >99%.

**Figure 1:**
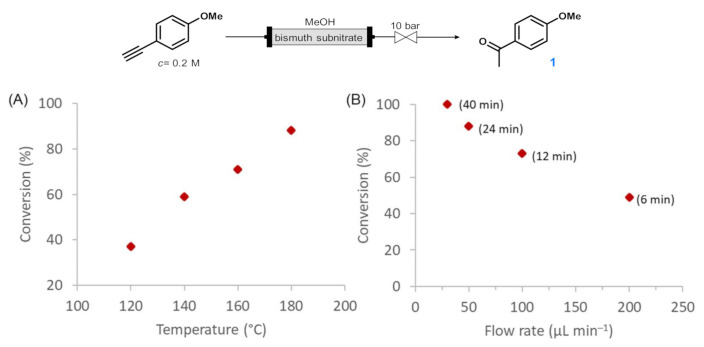
Investigation of the effects of various reaction conditions on the bismuth subnitrate-catalyzed hydration of *p*-methoxyphenylacetylene in a continuous flow packed-bed reactor. (**A**) Effects of the reaction temperature at 50 µL min^−1^ flow rate (24 min residence time). (**B**) Effects of the flow rate (residence time is shown in parenthesis) at 180 °C. The chemoselectivity towards acetophenone **1** was 100% in all reactions investigated.

With convincing results under batch conditions in our hands, we next attempted to translate the bismuth subnitrate-catalyzed alkyne hydration into a practical continuous flow process. Therefore, a simple setup was assembled that consisted of a stainless steel HPLC column as a catalyst bed and a 10-bar backpressure regulator (BPR), which enabled the safe overheating of the reaction mixture. The column encompassed 0.9 g of bismuth subnitrate as catalyst, and the 0.2-M alkyne solution in MeOH was continuously pumped by using an HPLC pump. Similarly, as in the batch experiments, the Markovnikov-type hydration of *p*-methoxyphenylacetylene was chosen as a model reaction. Firstly, the effect of the reaction temperature was investigated at a fixed flow rate of 50 µL min^−1^ (24-min residence time). A gradual improvement of conversion was found from 37% to 88%, with increasing temperatures in the range 120–180 °C (Figure 1A). Considering that in batch, a reaction time of 24 h was required for a quantitative reaction, 88% conversion within 12 min residence time should be regarded as a significant improvement. Next, the effects of different residence times were explored by employing different flow rates at 180 °C reaction temperature (Figure 1B). To our delight, upon decreasing the flow rate to 30 µL min^−1^ (40 min residence time), quantitative and selective hydration occurred into the desired acetophenone derivative. Shorter residence times gave lower conversions—for example, 49% in case of 6 min (200 µL min^−1^ flow rate). Gratifyingly, the formation of intermediary dimethyl acetal was not detected in any of these experiments.

After getting familiar with the effects of the key flow conditions, a range of alkynes were next submitted to bismuth subnitrate-catalyzed hydration at 180 °C and 30 µL min^−1^ flow rate (Table 6). Despite the fact that the 40 min residence time on the catalyst bed was reasonably shorter than the batch reaction times applied earlier (24–72 h), the flow reactions worked comparably well to the corresponding batch experiments. Excellent conversion (71–100%) and chemoselective acetophenone formation were observed in reactions of phenylacetylene and most of its mono- and multisubstituted derivatives investigated (entries 1–6). Similarly as in batch, only *p*-(tert-butyl)phenylacetylene gave moderate conversion among phenylacetylene derivatives investigated (entry 4). Gratifyingly, ethynylferrocene, 3-ethynylthiophene and ethyl propiolate, as well as dec-1-yne, were also tolerated well by the flow process, and their hydration furnished excellent conversions (90–100%) and selective formation of the desired methyl ketones (entries 7–10).

**Figure 2:**
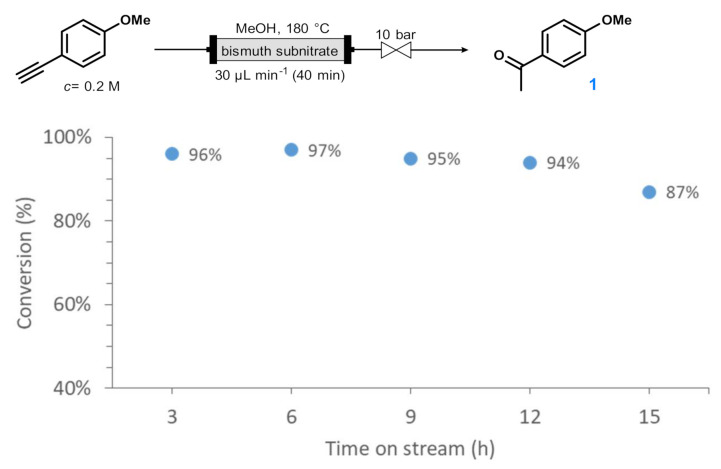
Time on stream vs. conversion curve for the bismuth subnitrate-catalyzed hydration of *p*-methoxyphenylacetylene in a continuous flow packed-bed reactor. Chemoselectivity towards acetophenone **1** was 100% in all points investigated.

Finally, the stability of the packed-bed system was tested. For this, the Markovnikov-type hydration of *p*-methoxyphenylacetylene was performed continuously for 15 h under optimum flow conditions (180 °C, 30 µL min^−1^ flow rate). During this period, five samples were taken and analyzed separately. To our delight, conversion was steady around 95% in the first 12 h of the experiment, and only a small decrease to 87% occurred in the last sample collected after 15 h (Figure 2). Importantly, the chemoselectivity towards acetophenone **1** was 100% in all samples investigated. As the result of this experiment, 725 mg of **1** was isolated, which corresponded to a yield of 89%. The bismuth content of the catalyst used during scale-out (including all washing cycles) showed a 3.1% decrease as compared with an unused batch of bismuth subnitrate, indicating the presence of some metal leaching under the comparatively harsh reaction conditions applied.

## 3. Materials and Methods

### 3.1. General Information

Reagents and materials were commercially available and used as received. Analytical thin-layer chromatography was performed on Merck silica gel 60 F254 plates (Sigma-Aldrich, Budapest, Hungary) and flash column chromatography on Merck silica gel 60 (Sigma-Aldrich, Budapest, Hungary). Compounds were visualized by means of UV or KMnO_4_. ^1^H NMR and ^13^C NMR spectra were recorded on a Bruker Avance NEO 500-MHz spectrometer (Bruker Corp., Billerica, MA, USA) equipped with a Prodigy BBO 5-mm CryoProbe, in CDCl_3_ as the solvent, with TMS as the internal standard. GC-MS analyses were performed on a Thermo Scientific Trace 1310 Gas Chromatograph (Thermo Fisher Scientific Corp., Waltham, MA, USA) coupled with a Thermo Scientific ISQ QD Single Quadrupole Mass Spectrometer (Thermo Fisher Scientific Corp., Waltham, MA, USA) using a Thermo Scientific TG-SQC column (15 m × 0.25 mm ID × 0.25 µ film). Measurement parameters were as follows: column oven temperature: from 50 to 300 °C at 15 °C min^−1^, injection temperature: 240 °C, ion source temperature: 200 °C; electrospray ionization: 70 eV, carrier gas: He at 1.5 mL min^‒1^, injection volume: 2 µL, split ratio: 1:33.3 and mass range: 50–500 *m*/*z*. Compound characterization data can be found in the Supplementary Materials. Bismuth content was measured by an Agilent 7900 ICP-MS in He gas mode. Argon was used as a carrier gas with 15 mL min^−1^ gas flow, while the He flow was 1 mL min^−1^. During the measurements, only the 209 *m*/*z* was monitored in Single Ion Mode.

### 3.2. General Procedure for Batch Reactions

A 2-mL reaction mixture containing 2 mmol of the appropriate alkyne, 0.3 mmol of bismuth subnitrate (15 mol%) and MeOH (or MeOH-*d*_4_) as the solvent was compiled in an oven-dried Schlenk tube equipped with a magnetic stir bar. The mixture was stirred for 24–72 h at 65 °C. After then, the mixture was cooled to room temperature, the catalyst was filtered off and concentrated in vacuo. The crude products were analyzed by NMR or GC-MS to determine the conversion and selectivity. If necessary, column chromatographic purification was carried out with mixtures of *n*-hexane/EtOAc as eluent. All products were thus achieved in purities ≥98%.

### 3.3. General Procedure for Flow Reactions

Reactions were performed in a home-made flow reactor consisting of a Jasco PU-2085 Plus HPLC pump (ABL&E-JASCO Hungary, Budapest, Hungary), a stainless-steel column as the catalyst bed (internal dimeter: 4.6 mm, length: 5 cm) and a 10-bar BPR purchased from IDEX. The parts of the system were connected with stainless-steel and PEEK capillary tubing (0.25-mm internal diameter, both) in combination with stainless-steel unions. The column was charged with 0.9 g of bismuth subnitrate using a small funnel and was sealed with compatible frits (2 mm pore size). For each reaction, a mixture consisting of the alkyne (c = 0.2 M) in MeOH was prepared and carefully homogenized, and the solution was pumped through the reactor under the appropriate conditions. In each run, 1–5 mL of product solution was collected, which was evaporated and analyzed by NMR or GC-MS to determine the conversion and selectivity. Between the two experiments, the system was washed for 20 min by pumping MeOH at a flow rate of 0.5 mL min^−1^. To obtain samples of the pure products, in some cases, column chromatographic purification was carried out with mixtures of *n*-hexane/EtOAc as the eluent. All products were thus obtained in purities ≥ 98%. The residence time on the catalyst bed was determined experimentally by pumping a dye solution. The elapsed time between the first contact of the dye with the column and the moment when the coloured solution appeared at the column outlet was measured.

## 4. Conclusions

Bismuth subnitrate was found as a simple, readily available and effective catalyst for the Markovnikov-type hydration of terminal acetylenes. Initially, the reactions were investigated under reasonably mild batch conditions, utilizing MeOH as solvent. The most important reaction conditions were thoroughly optimized in order to achieve high conversions and to eliminate intermediary dimethyl acetal formation. By switching the reaction medium from MeOH to MeOH-*d*_4_, a simple protocol was demonstrated to prepare trideuteromethyl ketones. Subsequently, a practical flow process was developed using a packed-bed apparatus. The effects of the reaction temperature and residence time were investigated carefully to achieve high conversions and selective methyl ketone formation in cases of diverse alkynes. Importantly, the utilization of continuous flow conditions enabled a marked chemical intensification as compared with the batch experiments and ensured time-efficient synthesis. The packed-bed system was found stable during 15 h of continuous experimentation. Due to the application of the inexpensive and nontoxic heterogeneous catalytic source, along with its high activity, high selectivity and, thus, low waste formation, the presented synthetic procedures possess marked benefits in terms of sustainability, as compared with earlier methodologies.

## Figures and Tables

**Table 1:** Investigation of various bismuth(III) compounds as catalysts in the Markovnikov-type hydration of *p*-methoxyphenylacetylene.

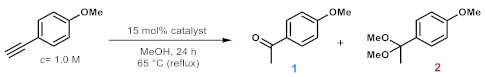				
Entry	Catalyst	Conversion (%) ^a^	Selectivity (%) ^a^	
			1	2
1	None	0	-	-
2	Bismuth subnitrate	100	100	0
3	Bi(OTf)_3_	100	88	12
4	BiBr_3_	45	92	8
5	Bi(OAc)_3_	0	-	-
6	Bi_2_O_3_	0	-	-

^a^ Determined by ^1^H NMR analysis of the crude product.

**Table 2:** Investigation of the effects of various reaction conditions on the bismuth subnitrate-catalyzed hydration of *p*-methoxyphenylacetylene.

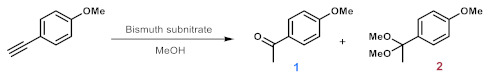							
Entry	Reaction Time (h)	Catalyst Loading (mol%)	C (M)	T (°C)	Conversion (%) ^a^	Selectivity (%) ^a^	
						1	2
1	24	15	1.0	65	100	100	0
2	12	15	1.0	65	73	100	0
3	6	15	1.0	65	41	100	0
4	3	15	1.0	65	18	100	0
5	1	15	1.0	65	6	100	0
6	24	10	1.0	65	92	100	0
7	24	5	1.0	65	79	88	12
8	24	2	1.0	65	62	71	29
9	24	15	1.0	25	3	100	0
10	24	15	2.0	65	73	92	8

^a^ Determined by ^1^H NMR analysis of the crude product.

**Table 3:** Investigation of the effects of various solvents on the bismuth subnitrate-catalyzed hydration of *p*-methoxyphenylacetylene.

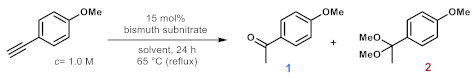				
Entry	Solvent	Conversion (%) ^a^	Selectivity (%) ^a^	
			1	2
1	MeOH	100	100	0
2	EtOH	14	100	0
3	*i*PrOH	traces	-	-
4	H_2_O	69	100	0
5	dry MeOH	94	92	8

^a^ Determined by ^1^H NMR analysis of the crude product.

**Table 4:** Exploring the bismuth subnitrate-catalyzed hydration of various alkynes under batch conditions.

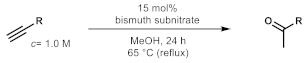				
Entry	Substrate	Product	Conversion (%) ^a,b^	Sel. (%) ^a^
1 ^c^	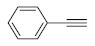	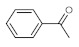	100 (98)	100
2	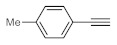	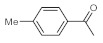	73	100
3	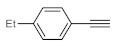	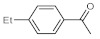	60	100
4	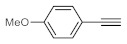	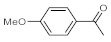	100 (98)	100
5 ^d^	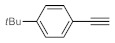	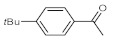	20	100
6	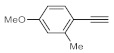	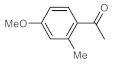	81	100
7	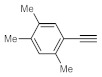	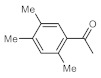	82	100
8	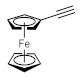	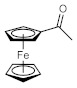	74	100
9 ^c^	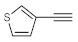	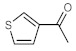	100	100
10 ^c^	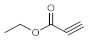	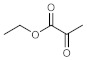	100	100

^a^ Determined by ^1^H NMR or GC-MS analysis of the crude product. ^b^ For representative examples, isolated yields are shown in parentheses. ^c^ 48-h reaction time. ^d^ 72-h reaction time.

**Table 5:** Exploring the bismuth subnitrate-catalyzed synthesis of various trideuteromethyl ketones.

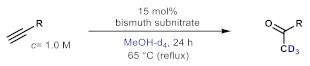					
Entry	Substrate	Product	Conversion (%) ^a^	Sel. (%) ^a^	D (%) ^a,b^
1	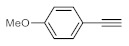	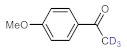	70	100	>99
2	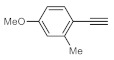	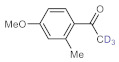	100	100	>99
3	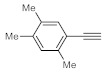	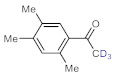	59	100	>99
4	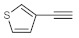	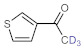	78	100	>99

^a^ Determined by ^1^H NMR analysis of the crude product. ^b^ Deuterium content (represent deuterium incorporation rate over incidental hydrogen incorporation).

**Table 6:** Exploring the bismuth subnitrate-catalyzed hydration of various alkynes under continuous flow conditions.

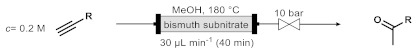				
Entry	Substrate	Product	Conversion (%) ^a^	Sel. (%) ^a^
1	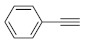	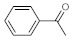	75	100
2	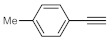	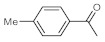	71	100
3	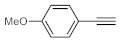	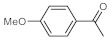	100	100
4	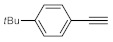	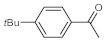	26	100
5	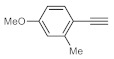	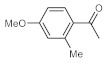	100	100
6	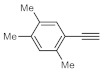	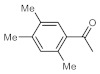	100	100
7	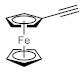	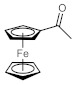	100	100
8	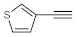	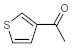	100	100
9	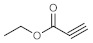	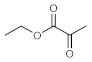	95	100
10	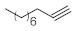	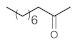	90	100

^a^ Determined by ^1^H NMR or GC-MS analysis of the crude product.

## Data Availability

All data supporting this study is available in the manuscript and in the Supplementary Materials.

## Supplementary Materials

The following are available online. Analytical data of the reaction products and the collection of NMR spectra.

## Sample Availability

Samples of the compounds are available from the authors.

## References

[1] Bryan M.C., Dunn P.J., Entwistle D., Gallou F., Koenig S.G., Hayler J.D., Hickey M.R., Hughes S., Kopach M.E., Moine G. (2018). Key Green Chemistry research areas from a pharmaceutical manufacturers' perspective revisited. Green Chem..

[2] Clarke C.J., Tu W.-C., Levers O., Bröhl A., Hallett J.P. (2018). Green and Sustainable Solvents in Chemical Processes. Chem. Rev..

[3] Sheldon R.A. (2017). The E factor 25 years on: The rise of green chemistry and sustainability. Green Chem..

[4] Egorova K.S., Ananikov V.P. (2016). Which Metals are Green for Catalysis? Comparison of the Toxicities of Ni, Cu, Fe, Pd, Pt, Rh, and Au Salts. Angew. Chem. Int. Ed..

[5] Erythropel H.C., Zimmerman J.B., de Winter T.M., Petitjean L., Melnikov F., Lam C.H., Lounsbury A.W., Mellor K.E., Janković N.Z., Tu Q. (2018). The Green ChemisTREE: 20 years after taking root with the 12 principles. Green Chem..

[6] Ollevier T. (2011). Bismuth-Mediated Organic Reactions. Topics in Current Chemistry.

[7] Ollevier T. (2013). New trends in bismuth-catalyzed synthetic transformations. Org. Biomol. Chem..

[8] Nordberg G.F. (2015). Handbook on the Toxicology of Metals.

[9] Matano Y. (2001). Organobismuth Chemistry.

[10] Mohan R. (2010). Green bismuth. Nat. Chem..

[11] Ondet P., Lemière G., Duñach E. (2017). Cyclisations Catalysed by Bismuth(III) Triflate. Eur. J. Org. Chem..

[12] Salvador J.A.R., Ppinto R.M.A., Silvestre S.M. (2009). Recent Advances of Bismuth(III) Salts in Organic Chemistry: Application to the Synthesis of Aliphatics, Alicyclics, Aromatics, Amino Acids and Peptides, Terpenes and Steroids of Pharmaceutical Interest. Mini-Rev. Org. Chem..

[13] Ruimao H. (2008). Recent Advances in Bismuth-Catalyzed Organic Synthesis. Curr. Org. Synth..

[14] Gaspard-Iloughmane H., Le Roux C. (2004). Bismuth(III) Triflate in Organic Synthesis. Eur. J. Org. Chem..

[15] Leonard N.M., Wieland L.C., Mohan R.S. (2002). Applications of bismuth(III) compounds in organic synthesis. Tetrahedron.

[16] Bothwell J.M., Krabbe S.W., Mohan R.S. (2011). Applications of bismuth(III) compounds in organic synthesis. Chem. Soc. Rev..

[17] Rueping M., Nachtsheim B.J., Ollevier T. (2012). Bismuth Salts in Catalytic Alkylation Reactions. Bismuth-Mediated Organic Reactions.

[18] Rueping M., Nachtsheim B.J., Ieawsuwan W. (2006). An Effective Bismuth-Catalyzed Benzylation of Arenes and Heteroarenes. Adv. Synth. Catal..

[19] Rueping M., Nachtsheim B.J., Kuenkel A. (2007). Efficient Metal-Catalyzed Direct Benzylation and Allylic Alkylation of 2,4-Pentanediones. Org. Lett..

[20] Ötvös S.B., Szécsényi Z., Fülöp F. (2019). Bismuth(III)-Catalyzed Hydration of Terminal Alkynes: Sustainable Synthesis of Methyl Ketones in Batch and Flow. ACS Sustain. Chem. Eng..

[21] Sun H.-B., Li B., Hua R., Yin Y. (2006). An Efficient and Selective Hydroarylation of Styrenes with Electron-Rich Arenes, Catalyzed by Bismuth(III) Chloride and Affording Markovnikov Adducts. Eur. J. Org. Chem..

[22] Rueping M., Nachtsheim B.J., Sugiono E. (2010). Direct Catalytic Benzylation of Hydroxycoumarin—Efficient Synthesis of Warfarin Derivatives and Analogues. Synlett.

[23] Qin H., Yamagiwa N., Matsunaga S., Shibasaki M. (2006). Bismuth-Catalyzed Intermolecular Hydroamination of 1,3-Dienes with Carbamates, Sulfonamides, and Carboxamides. J. Am. Chem. Soc..

[24] Wei H., Qian G., Xia Y., Li K., Li Y., Li W. (2007). BiCl_3_-Catalyzed Hydroamination of Norbornene with Aromatic Amines. Eur. J. Org. Chem..

[25] Ötvös S.B., Mészáros R., Varga G., Kocsis M., Kónya Z., Kukovecz Á., Pusztai P., Sipos P., Pálinkó I., Fülöp F. (2018). A mineralogically-inspired silver–bismuth hybrid material: An efficient heterogeneous catalyst for the direct synthesis of nitriles from terminal alkynes. Green Chem..

[26] Beller M., Seayad J., Tillack A., Jiao H. (2004). Catalytic Markovnikov and anti-Markovnikov Functionalization of Alkenes and Alkynes: Recent Developments and Trends. Angew. Chem. Int. Ed..

[27] Hintermann L., Labonne A. (2007). Catalytic Hydration of Alkynes and Its Application in Synthesis. Synthesis.

[28] Alabugin I.V., Gonzalez-Rodriguez E., Kawade R.K., Stepanov A.A., Vasilevsky S.F. (2019). Alkynes as Synthetic Equivalents of Ketones and Aldehydes: A Hidden Entry into Carbonyl Chemistry. Molecules.

[29] Dorel R., Echavarren A.M. (2015). Gold(I)-Catalyzed Activation of Alkynes for the Construction of Molecular Complexity. Chem. Rev..

[30] Fang G., Bi X. (2015). Silver-catalysed reactions of alkynes: Recent advances. Chem. Soc. Rev..

[31] Marion N., Ramón R.S., Nolan S.P. (2009). [(NHC)Au^I^]-Catalyzed Acid-Free Alkyne Hydration at Part-per-Million Catalyst Loadings. J. Am. Chem. Soc..

[32] Xu Y., Hu X., Shao J., Yang G., Wu Y., Zhang Z. (2015). Hydration of alkynes at room temperature catalyzed by gold(I) isocyanide compounds. Green Chem..

[33] Rao K.T.V., Prasad P.S.S., Lingaiah N. (2012). Solvent-free hydration of alkynes over a heterogeneous silver exchanged silicotungstic acid catalyst. Green Chem..

[34] Thuong M.B.T., Mann A., Wagner A. (2012). Mild chemo-selective hydration of terminal alkynes catalysed by AgSbF_6_. Chem. Commun..

[35] Trentin F., Chapman A.M., Scarso A., Sgarbossa P., Michelin R.A., Strukul G., Wass D.F. (2012). Platinum(II) Diphosphinamine Complexes for the Efficient Hydration of Alkynes in Micellar Media. Adv. Synth. Catal..

[36] Liu X., Liu L., Wang Z., Fu X. (2015). Visible light promoted hydration of alkynes catalyzed by rhodium(III) porphyrins. Chem. Commun..

[37] Tachinami T., Nishimura T., Ushimaru R., Noyori R., Naka H. (2013). Hydration of Terminal Alkynes Catalyzed by Water-Soluble Cobalt Porphyrin Complexes. J. Am. Chem. Soc..

[38] Mainkar P.S., Chippala V., Chegondi R., Chandrasekhar S. (2016). Ruthenium(II)-Catalyzed Hydration of Terminal Alkynes in PEG-400. Synlett.

[39] Keogan D.M., Griffith D.M. (2014). Current and Potential Applications of Bismuth-Based Drugs. Molecules.

[40] Lazarini F. (1981). Thermal dehydration of some basic bismuth nitrates. Thermochim. Acta.

[41] Reddy Y.T., Rajitha B., Reddy P.N., Kumar B.S., Rao V.P. (2004). Bismuth Subnitrate Catalyzed Efficient Synthesis of 3,4-Dihydropyrimidin-2(1H)-Ones: An Improved Protocol for the Biginelli Reaction. Synth. Commun..

[42] Wu S., Dai W., Yin S., Li W., Au C.-T. (2008). Bismuth Subnitrate as an Efficient Heterogeneous Catalyst for Acetalization and Ketalization of Carbonyl Compounds with Diols. Catal. Lett..

[43] Tanimu A., Jaenicke S., Alhooshani K. (2017). Heterogeneous catalysis in continuous flow microreactors: A review of methods and applications. Chem. Eng. J..

[44] Ciriminna R., Pagliaro M., Luque R. (2021). Heterogeneous catalysis under flow for the 21st century fine chemical industry. Green Energy Environ..

[45] Liu X., Unal B., Jensen K.F. (2012). Heterogeneous catalysis with continuous flow microreactors. Catal. Sci. Technol..

[46] Frost C.G., Mutton L. (2010). Heterogeneous catalytic synthesis using microreactor technology. Green Chem..

[47] Munirathinam R., Huskens J., Verboom W. (2015). Supported Catalysis in Continuous-Flow Microreactors. Adv. Synth. Catal..

[48] Yoo W.-J., Ishitani H., Saito Y., Laroche B., Kobayashi S. (2020). Reworking Organic Synthesis for the Modern Age: Synthetic Strategies Based on Continuous-Flow Addition and Condensation Reactions with Heterogeneous Catalysts. J. Org. Chem..

[49] Masuda K., Ichitsuka T., Koumura N., Sato K., Kobayashi S. (2018). Flow fine synthesis with heterogeneous catalysts. Tetrahedron.

[50] Ötvös S.B., Pericàs M.A., Kappe C.O. (2019). Multigram-scale flow synthesis of the chiral key intermediate of (−)-paroxetine enabled by solvent-free heterogeneous organocatalysis. Chem. Sci..

[51] Ötvös S.B., Llanes P., Pericàs M.A., Kappe C.O. (2020). Telescoped Continuous Flow Synthesis of Optically Active γ-Nitrobutyric Acids as Key Intermediates of Baclofen, Phenibut, and Fluorophenibut. Org. Lett..

[52] Mándity I.M., Ötvös S.B., Fülöp F. (2015). Strategic Application of Residence-Time Control in Continuous-Flow Reactors. ChemistryOpen.

[53] Plutschack M.B., Pieber B., Gilmore K., Seeberger P.H. (2017). The Hitchhiker’s Guide to Flow Chemistry. Chem. Rev..

[54] Akwi F.M., Watts P. (2018). Continuous flow chemistry: Where are we now? Recent applications, challenges and limitations. Chem. Commun..

[55] Rogers L., Jensen K.F. (2019). Continuous manufacturing—The Green Chemistry promise?. Green Chem..

[56] Hou S., Yang H., Cheng B., Zhai H., Li Y. (2017). Cobaloxime-catalyzed hydration of terminal alkynes without acidic promoters. Chem. Commun..

[57] Mazzone G., Russo N., Sicilia E. (2012). Homogeneous Gold Catalysis: Hydration of 1,2-Diphenylacetylene with Methanol in Aqueous Media. A Theoretical Viewpoint. Organometallics.

[58] Wang H., Hussain A.A., Pyrek J.S., Goodman J., Wedlund P.J. (2004). Assay for nipecotic acid in small blood samples by gas chromatography–mass spectroscopy. J. Pharmaceut. Biomed..

[59] Sanderson K. (2009). Big interest in heavy drugs. Nature.

[60] Marcus D.M., McLachlan K.A., Wildman M.A., Ehresmann J.O., Kletnieks P.W., Haw J.F. (2006). Experimental Evidence from H/D Exchange Studies for the Failure of Direct C-C Coupling Mechanisms in the Methanol-to-Olefin Process Catalyzed by HSAPO-34. Angew. Chem. Int. Ed..

[61] Atzrodt J., Derdau V., Kerr W.J., Reid M. (2018). Deuterium- and Tritium-Labelled Compounds: Applications in the Life Sciences. Angew. Chem. Int. Ed..

[62] Atzrodt J., Derdau V., Fey T., Zimmermann J. (2007). The Renaissance of H/D Exchange. Angew. Chem. Int. Ed..

[63] Coumbarides G.S., Dingjan M., Eames J., Flinn A., Northen J. (2006). An efficient laboratory synthesis of α-deuteriated profens. J. Label. Compd. Radiopharm..

[64] Erdogan G., Grotjahn D.B. (2009). Mild and Selective Deuteration and Isomerization of Alkenes by a Bifunctional Catalyst and Deuterium Oxide. J. Am. Chem. Soc..

[65] Zhan M., Zhang T., Huang H., Xie Y., Chen Y. (2014). A simple method for α-position deuterated carbonyl compounds with pyrrolidine as catalyst. J. Label. Compd. Radiopharm..

