# Supplementary material for: Bismuth Subnitrate-Catalyzed Markovnikov-Type Alkyne Hydrations under Batch and Continuous Flow Conditions
Source: Molecules. 2021 May 12;26(10):2864. doi: 10.3390/molecules26102864 (PMC8151695; doi:10.3390/molecules26102864)
Supplement: Supplementary file 1 [file molecules-26-02864-s001.zip › molecules-1215721-SI.pdf]

Supporting Information

for

Bismuth Subnitrate-Catalyzed Markovnikov-type Alkyne Hydrations under Batch and Continuous Flow Conditions

Zsanett Szécsényi [1], Ferenc Fülöp [1,2,] and Sándor B. Ötvös [2,3,*]*

[1] *Institute of Pharmaceutical Chemistry, University of Szeged, Interdisciplinary Excellence Center, Eötvös u. 6, Szeged, H-6720 Hungary.*

[2] *MTA-SZTE Stereochemistry Research Group, Hungarian Academy of Sciences, Interdisciplinary Excellence Center, Eötvös u. 6, Szeged, H-6720.*

[3] *Institute of Chemistry, University of Graz, NAWI Graz, Heinrichstrasse 28, Graz, A-8010 Austria.*

**Correspondence: fulop@pharm.u-szeged.hu (FF), sandor.oetvoes@uni-graz.at (SBO)*

Table of Contents

1. **Analytical data of reaction products**..3
2. **Collection of NMR spectra**..8
3. **References**..30

1. Analytical data of reaction products

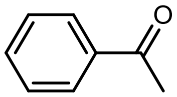

Acetophenone:

^{1}H NMR (500 MHz, $CDCl_3$) δ 7.97 (d, *J* = 7.0 Hz, 2H), 7.57 (t, *J* = 7.4 Hz, 1H), 7.47 (t, *J* = 7.8 Hz, 2H), 2.62 (s, 3H) ppm
^{13}C NMR (126 MHz, $CDCl_3$) δ 198.4, 137.3, 133.3, 128.7, 128.5, 26.8 ppm
MS (EI) m/z = 120.08, 105.04, 77.06, 51.05
NMR data is in agreement with the published data.[1]
(Column chromatography in *n*-hexane/EtOAc 4:1 as eluent.)

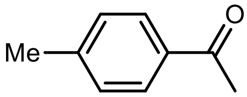

4-Methyl-acetophenone:

^{1}H NMR (500 MHz, $CDCl_3$) 7.86 (d, *J* =8.1 Hz, 2H), 7.26 (d, *J* =8.0 Hz, 2H), 2.58 (s, 3H), 2.41 (s, 3H) ppm
^{13}C NMR (126 MHz, $CDCl_3$) 197.9, 143.9, 134.7, 129.3, 128.5, 26.6, 21.7 ppm
MS (EI) m/z = 134.08, 119.07, 91.06, 77.05, 65.04
NMR data is in agreement with the published data.[2]
(Column chromatography in *n*-hexane/EtOAc 2:1 as eluent.)

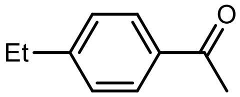

4-Ethyl-acetophenone:

^{1}H NMR (500 MHz, $CDCl_3$) δ 7.89 (d, J = 8.3 Hz, 2H), 7.28 (d, J = 8.0 Hz, 2H), 2.71 (q, J = 7.6 Hz, 2H), 2.58 (s, 3H), 1.26 (t, J = 7.6 Hz, 3H)
^{13}C NMR (126 MHz, $CDCl_3$) 197.9, 150.1, 134.9, 128.6, 128.1, 29.0, 26.6, 15.2 ppm
MS (EI) m/z 148.08, 133.07, 105.04, 91.05, 77.06, 63.07
NMR data is in agreement with the published data.[3]
(Column chromatography in *n*-hexane/EtOAc 2:1 as eluent.)

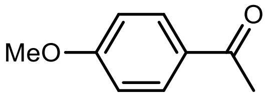

4-Methoxy-acetophenone:

^{1}H NMR (500 MHz, $CDCl_3$) δ 7.94 (d, J = 8.9 Hz, 2H), 6.93 (d, J = 9.0 Hz, 2H), 3.87 (s, 3H), 2.56 (s, 3H) ppm
^{13}C NMR (126 MHz, $CDCl_3$) 196.8, 163.5, 130.6, 130.3, 113.7, 55.5, 26.4 ppm
MS (EI) m/z = 150.08, 135.06, 107.05, 92.03, 77.04, 63.03
NMR data is in agreement with the published data.[2]

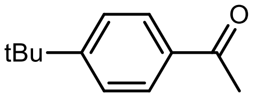

4-*tert*Buthyl-acetophenone:

^{1}H NMR (500 MHz, $CDCl_3$) δ 7.90 (d, *J* = 8.5 Hz, 2H), 7.48 (d, *J* = 8.5 Hz, 2H), 2.58 (s, 3H), 1.34 (s, 9H) ppm
^{13}C NMR (126 MHz, $CDCl_3$) δ 197.9, 156.8, 134.6, 128.3, 125.5, 35.1, 31.1, 26.6 ppm
MS (EI) *m/z* = 176.12, 161.11, 146.09, 133.09, 115.09, 105.07, 91.06, 77.06, 51.03
NMR data is in agreement with the published data.[2]
(Column chromatography in *n*-hexane/EtOAc 9:1 as eluent.)

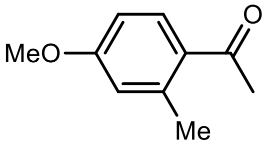

4-Methoxy-2-methylacetophenone

^{1}H NMR (500 MHz, $CDCl_3$) δ 7.76 (d, *J* = 8.3 Hz, 1H), 6.75 (d, *J* = 8.3 Hz, 2H), 3.84 (s, 3H), 2.57 (s, 3H), 2.55 (s, 3H) ppm
^{13}C NMR (126 MHz, $CDCl_3$) δ 199.5, 162.0, 142.3, 132.6, 130.0, 117.5, 110.6, 55.3, 29.1, 22.7 ppm
MS (EI) m/z = 164.10, 149.08, 121.07, 105.06, 91.07, 77.07, 51.03
NMR data is in agreement with the published data.[5,6]
(Column chromatography in *n*-hexane/EtOAc 1:1 as eluent.)

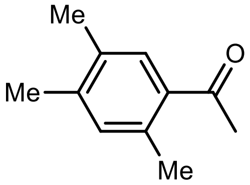

2,4,5-Trimethylacetophenone

^{1}H NMR (500 MHz, $CDCl_3$) δ 7.48 (s, 1H), 6.99 (s, 1H), 2.55 (s, 3H), 2.47 (s, 3H), 2.26 (s, 3H), 2.25 (s, 3H) ppm

^{13}C NMR (126 MHz, $CDCl_3$) δ 201.2, 140.8, 136.2, 135.0, 133.7, 133.5, 131.1, 29.4, 21.3, 19.7, 19.3 ppm
MS (EI) m/z = 162.12, 147.09, 119.10, 103.08, 91.06, 77.04, 51.03
NMR data is in agreement with the published data.[7]
(Column chromatography in *n*-hexane/EtOAc 9:1 as eluent.)

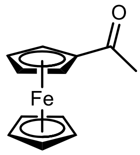

Acetylferrocene:

^{1}H NMR (500 MHz, $CDCl_3$) δ 4.76 (t, J = 2.0 Hz, 2H), 4.50 – 4.47 (m, 2H), 4.19 (s, 5H), 2.38 (s, 3H) ppm
^{13}C NMR (126 MHz, $CDCl_3$) δ 202.2, 79.3, 72.4, 69.9, 69.7, 27.5 ppm
MS (EI) m/z = 228.03, 213.01, 184.99, 163.01, 129.08, 120.97, 93.97, 80.95, 65.05, 55.93
NMR data is in agreement with the published data.[9]
(Column chromatography in *n*-hexane/EtOAc 2:1 as eluent.)

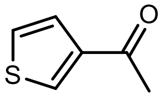

3-Acetylthiophene:

^{1}H NMR (500 MHz, $CDCl_3$) δ 8.04 (dd, *J* = 3.0, 1.3 Hz, 1H), 7.54 (dd, *J* = 5.1, 1.3 Hz, 1H), 7.31 (dd, *J* = 5.2, 2.9 Hz, 1H), 2.53 (s, 3H) ppm
^{13}C NMR (126 MHz, CDCl3) δ 192.3, 142.6, 132.4, 127.0, 126.4, 27.6 ppm
MS (EI) m/z = 126.02, 111.00, 82.99
NMR data is in agreement with the published data.[8]

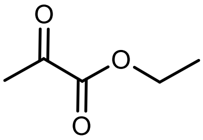

Ethyl-pyruvate:

^{1}H NMR (500 MHz, $CDCl_3$) δ 4.32 (q, J = 7.2 Hz, 2H), 2.47 (d, J = 1.1 Hz, 3H), 1.37 (d, J = 14.4 Hz, 3H) ppm
^{13}C NMR (126 MHz, $CDCl_3$) δ 191.85, 160.72, 62.2, 26.4, 13.8 ppm
MS (EI) m/z = 116.1, 73.1, 61.1
NMR data is in agreement with the published data.[10]
(Column chromatography in *n*-hexane/EtOAc 1:1 as eluent.)

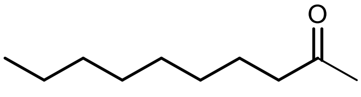

2-Decanone:

1H NMR (500 MHz, $CDCl_3$) δ 2.42 (t, J = 7.5 Hz, 2H), 2.13 (s, 3H), 1.55 (dd, J = 16.8, 10.9 Hz, 2H), 1.28 – 1.26 (m, 10H), 0.88 (t, J = 6.8 Hz, 3H).

^{13}C NMR (126 MHz, $CDCl_3$) 209.6, 43.8, 31.8, 29.9. 29.4, 29.2, 29.1, 23.9, 22.6, 14.1 ppm
MS (EI) *m/z* = 156.16, 141.15, 113.11, 96.12, 85.09, 71.07, 58.05
NMR data is in agreement with the published data.[11]
(Column chromatography in *n*-hexane/EtOAc 1:2 as eluent.)

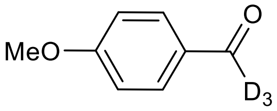

1-(4-methoxyphenyl)ethan-1-one-2,2,2-*d3*:

^{1}H NMR (500 MHz, $CDCl_3$) δ 7.91 (d, 2H), 6.91 (d, 2H), 3.85 (s, 3H) ppm
^{13}C NMR (126 MHz, $CDCl_3$) δ 196.8, 163.4, 130.5, 130.2, 113.5, 55.4, 25.5 ppm
MS (EI) *m/z* = 153.14, 135.08, 107.10, 92.04, 77.08, 64.08
^{1}H NMR data is in agreement with the published data.[12]
(Column chromatography in *n*-hexane/EtOAc 4:1 as eluent.)

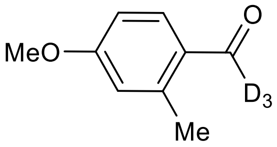

1-(4-methoxy-2-methylphenyl)ethan-1-one-2,2,2-*d3*:

^{1}H NMR (500 MHz, $CDCl_3$) δ 7.74 (d, 1H), 6.75 (d, 2H), 3.84 (s, 3H), 2.57 (s, 3H) ppm
^{13}C NMR (126 MHz, $CDCl_3$) δ 199.5, 161.9, 142.2, 132.4, 130.0, 117.5, 110.5, 55.3, 28.1, 22.4 ppm
MS (EI) *m/z* = 167.15, 149.11, 91.11, 77.11

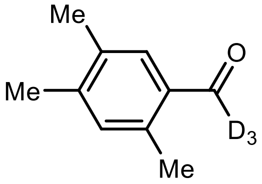

1-(2,4,5-trimethylphenyl)ethan-1-one-2,2,2-*d3*:

^{1}H NMR (500 MHz, $CDCl_3$) δ 7.47 (s, 1H), 7.00 (s, 1H), 2.47 (s, 3H), 2.27 (s, 6H) ppm

^{13}C NMR (126 MHz, $CDCl_3$) δ 200.9, 140.7, 136.1, 135.2, 133.6, 133.5, 131.0, 31.5, 21.0, 19.5, 19.1 ppm
MS (EI) *m/z* = 165.19, 147.13, 119.15, 91.10, 77.10
(Column chromatography in *n*-hexane/EtOAc 9:1 as eluent.)

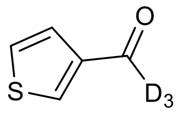

1-(thiophen-3-yl)ethan-1-one-2,2,2-*d3*:

^{1}H NMR (500 MHz, $CDCl_3$) δ 8.03 (dd, *J* = 3.0, 1.3 Hz, 1H), 7.54 (dd, J=5.1, Hz, 1H), 7.31 (dd, J= 5.1, 2.9 Hz, 1 H) ppm
^{13}C NMR (126 MHz, $CDCl_3$) δ 192.4, 142.6, 132.3, 126.9, 126.3, 29.7 ppm
MS (EI) *m/z* = 129.06, 128.04, 111.01, 83.08
(Column chromatography in *n*-hexane/EtOAc 2:1 as eluent.)

2. Collection of NMR spectra

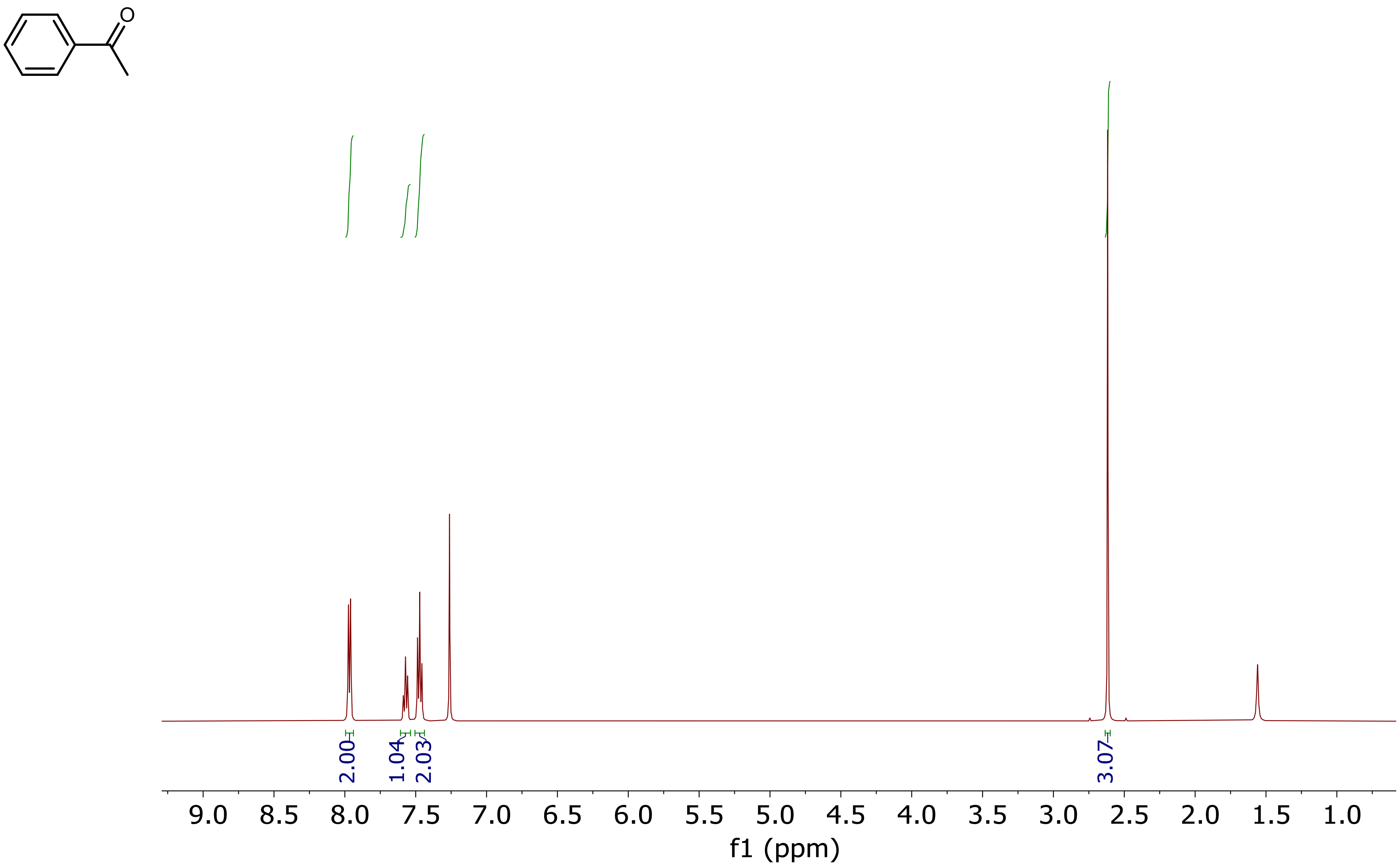

Figure S1. ^{1}H NMR spectrum of acetophenone in $CDCl_3$.

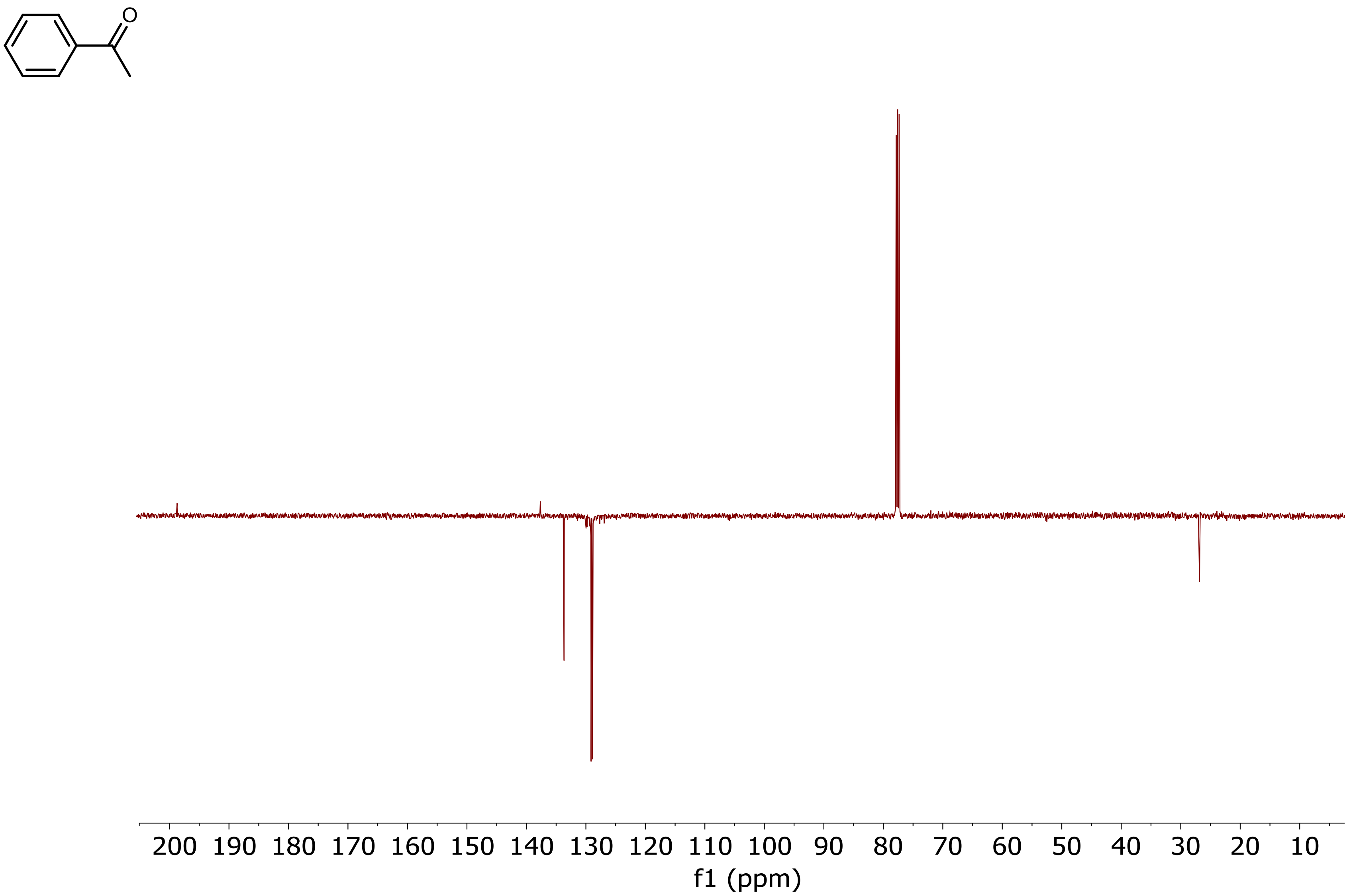

Figure S2. ^{13}C NMR spectrum of acetophenone in $CDCl_3$.

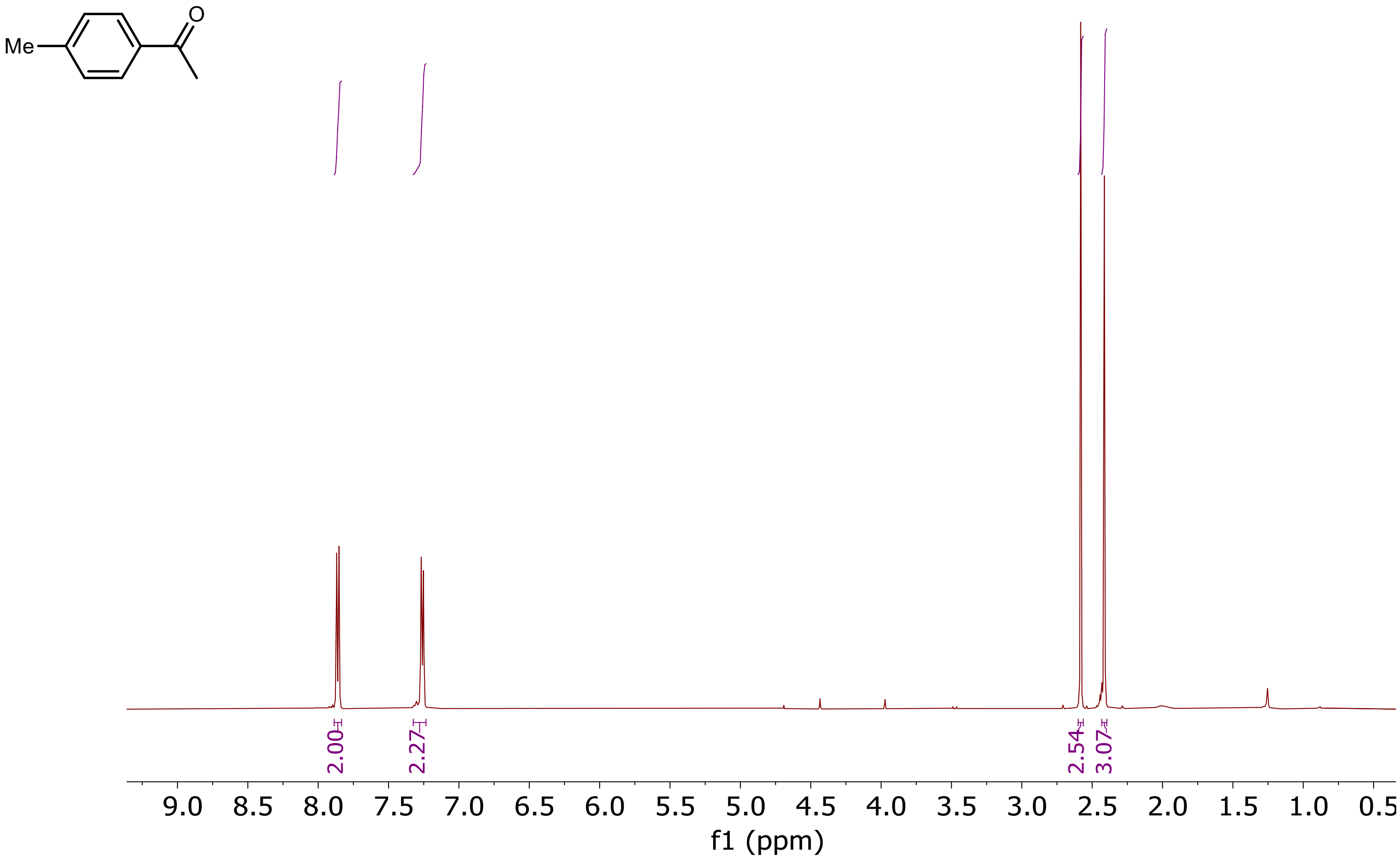

Figure S3. ^{1}H NMR spectrum of 4-methylacetophenone in $CDCl_3$.

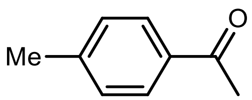

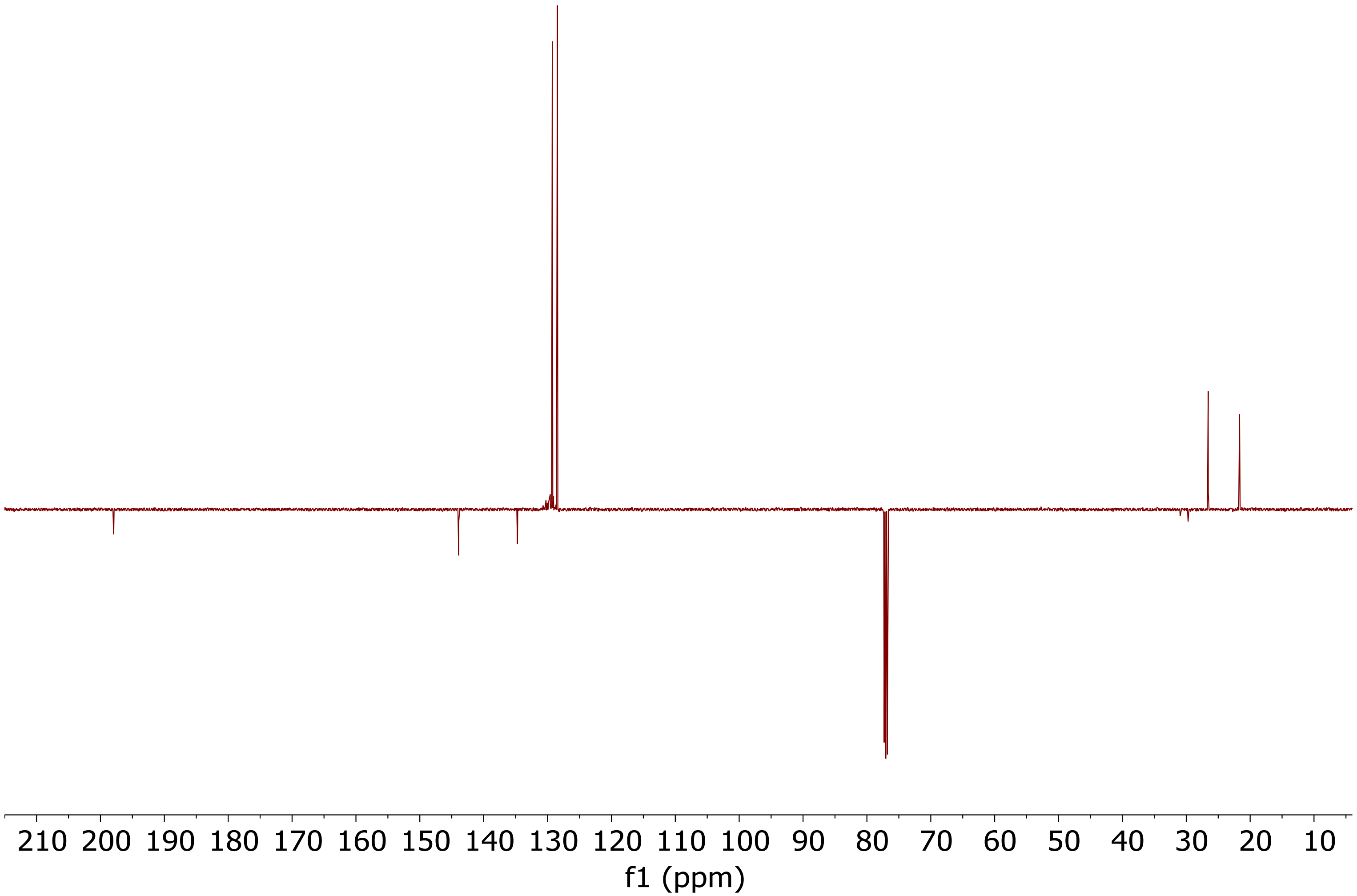

Figure S4. ^{13}C NMR spectrum of 4-methylacetophenone in $CDCl_3$.

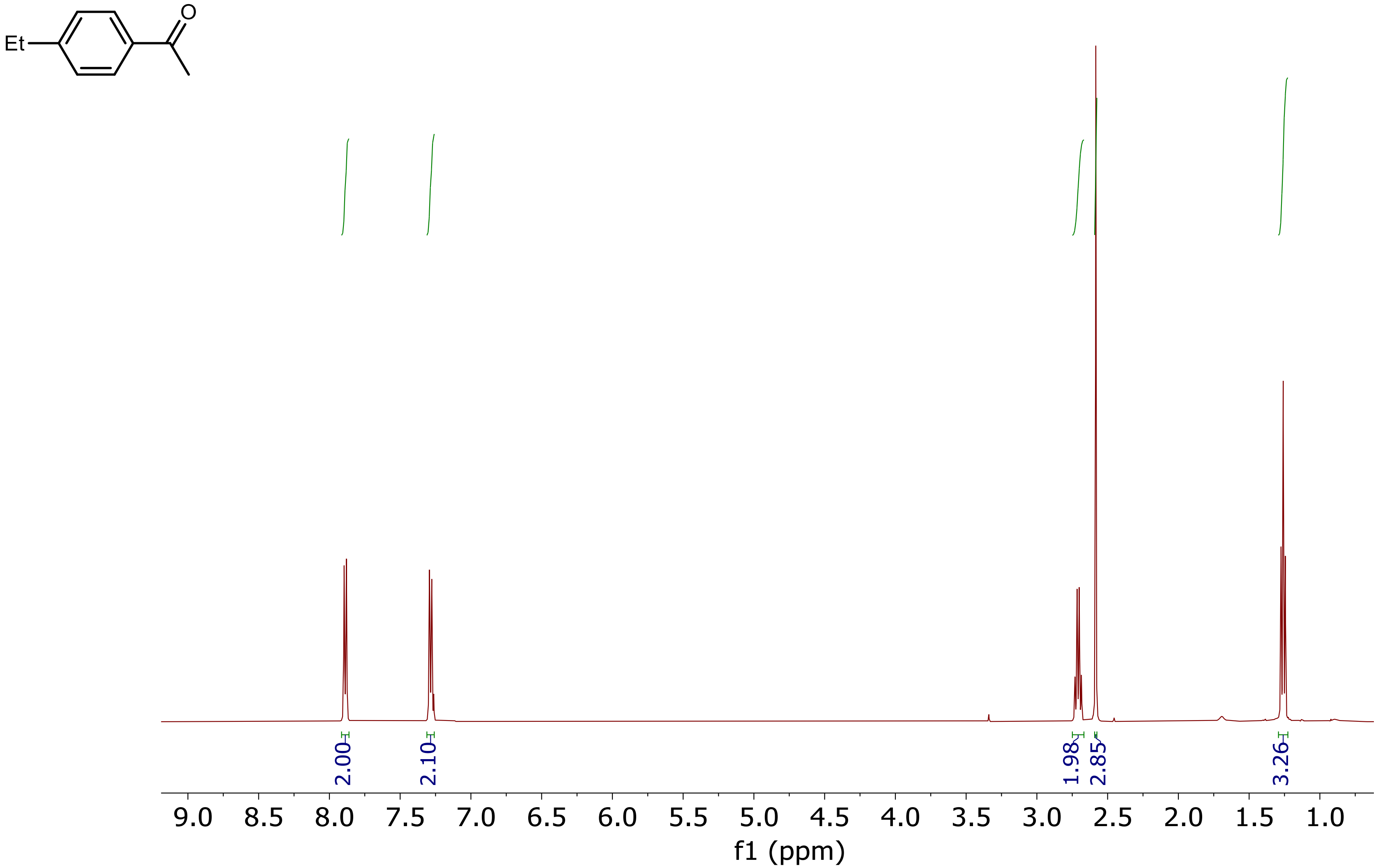

Figure S5. ^{1}H NMR spectrum of 4-ethylacetophenone in $CDCl_3$**.**

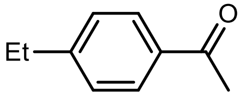

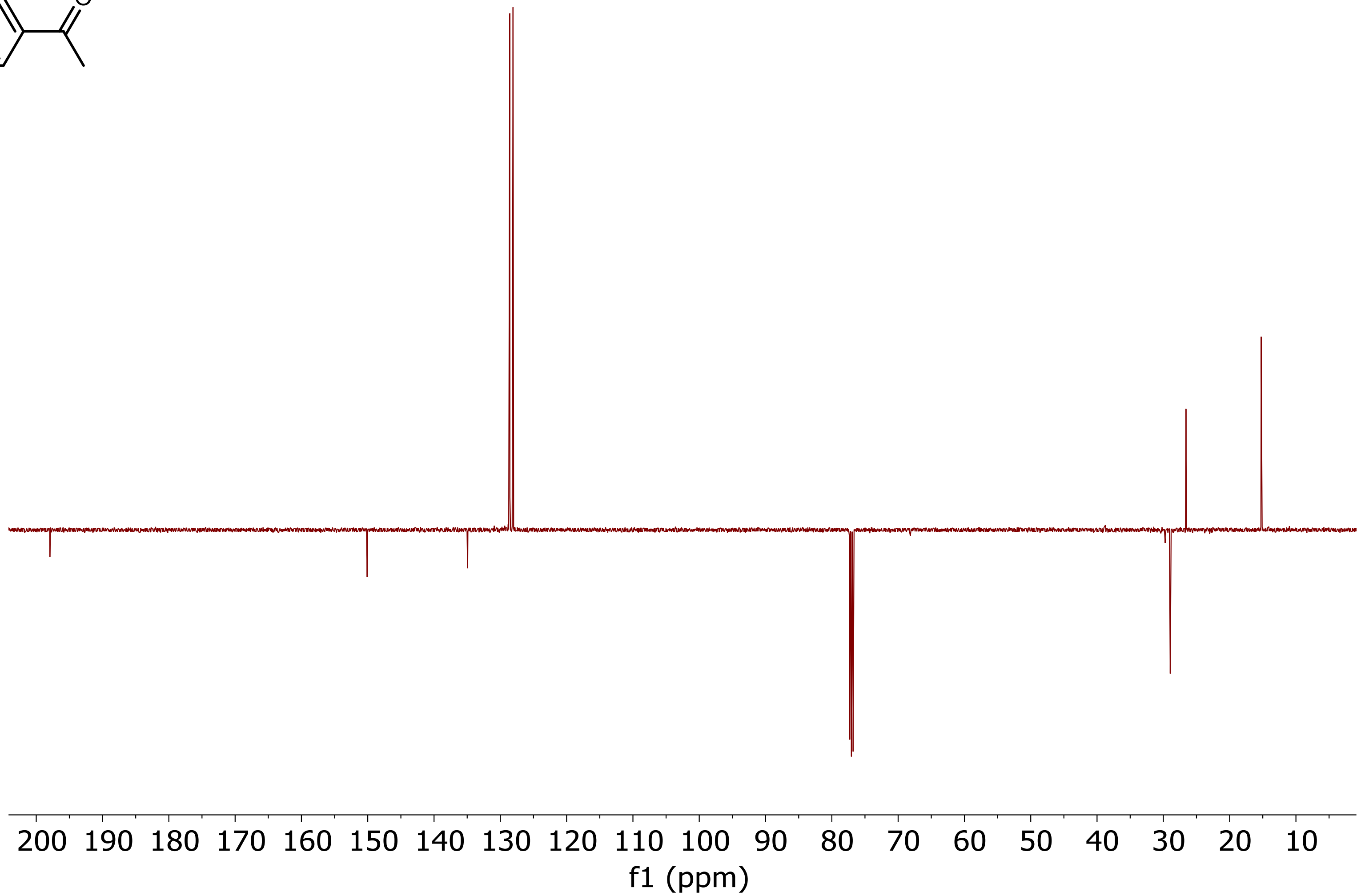

Figure S6. ^{13}C NMR spectrum of 4-ethylacetophenone in $CDCl_3$**.**

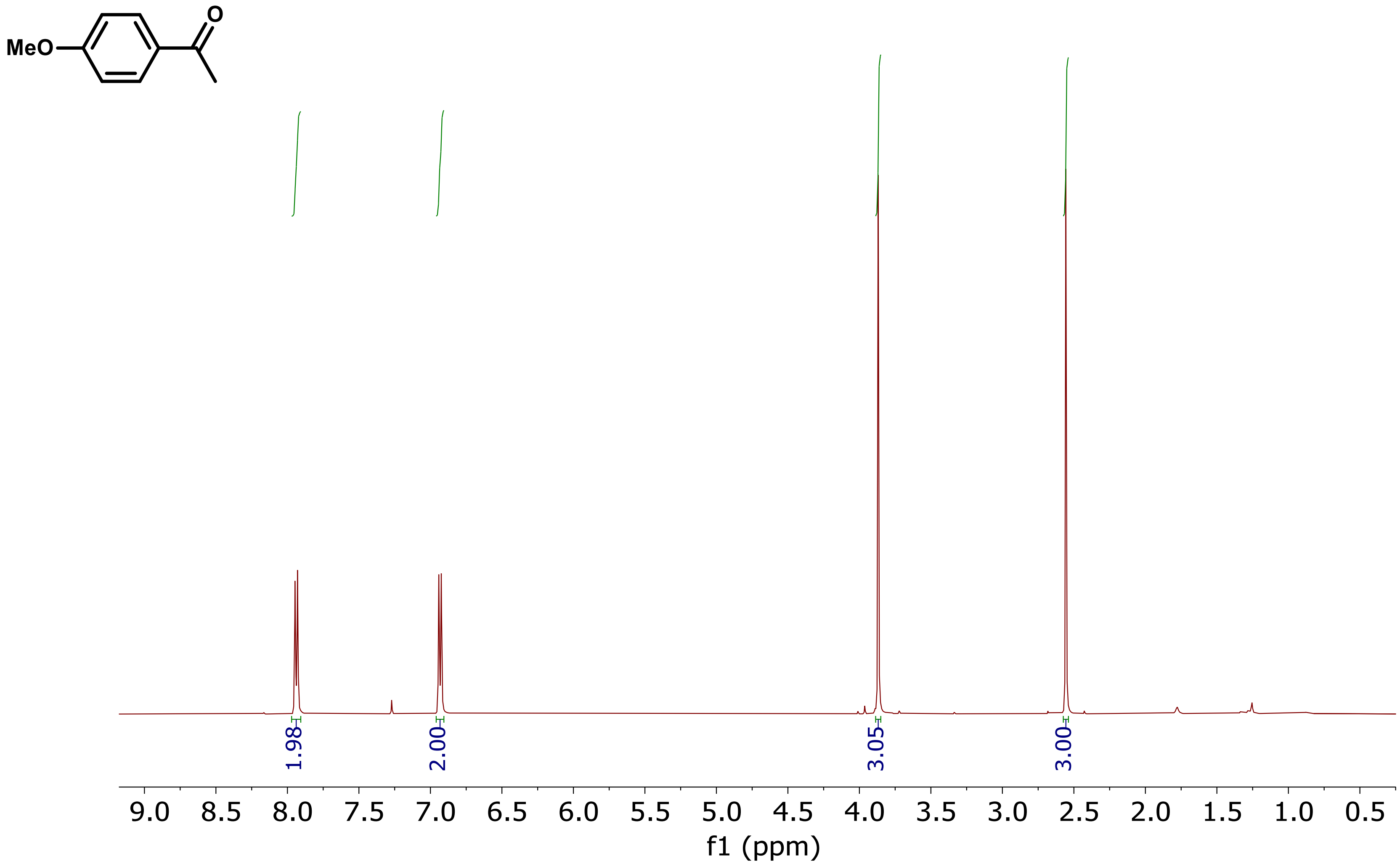

Figure S7. ^{1}H NMR spectrum of 4-methoxyacetophenone in $CDCl_3$.

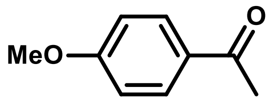

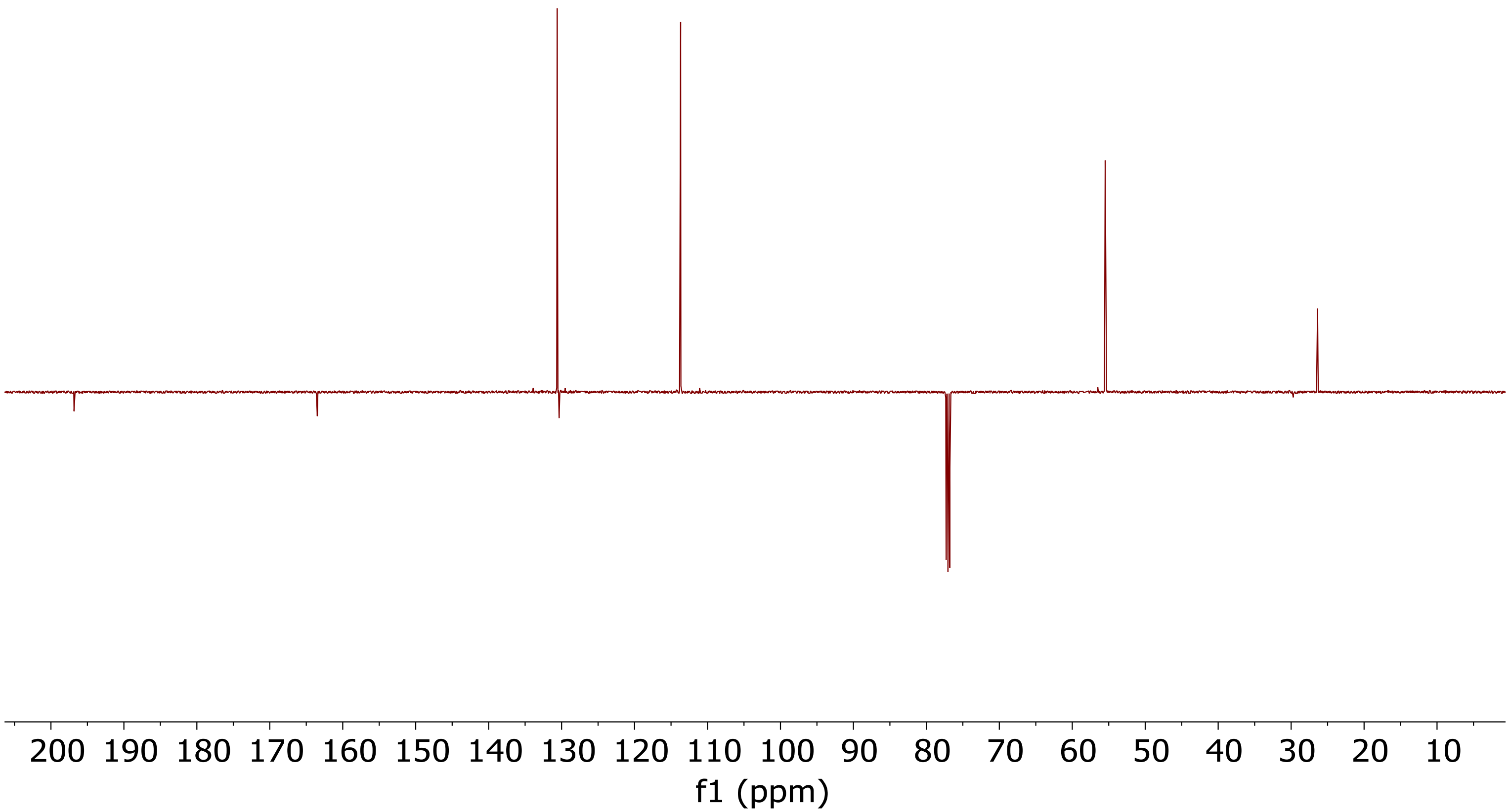

Figure S8. ^{13}C NMR spectrum of 4-methoxyacetophenone in $CDCl_3$.

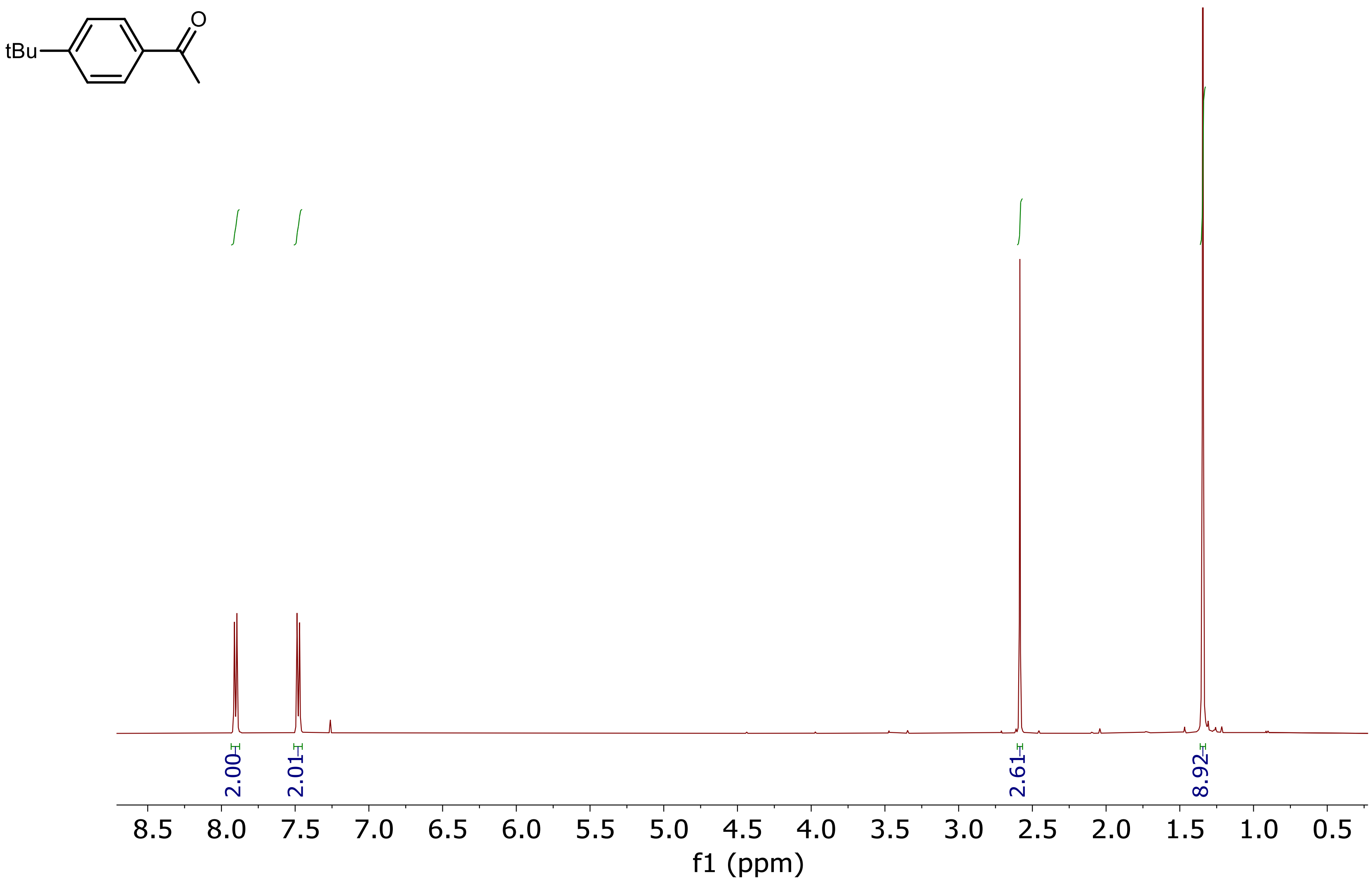

Figure S9. ^{1}H NMR spectrum of 4-*tert*buthylacetophenone in $CDCl_3$..

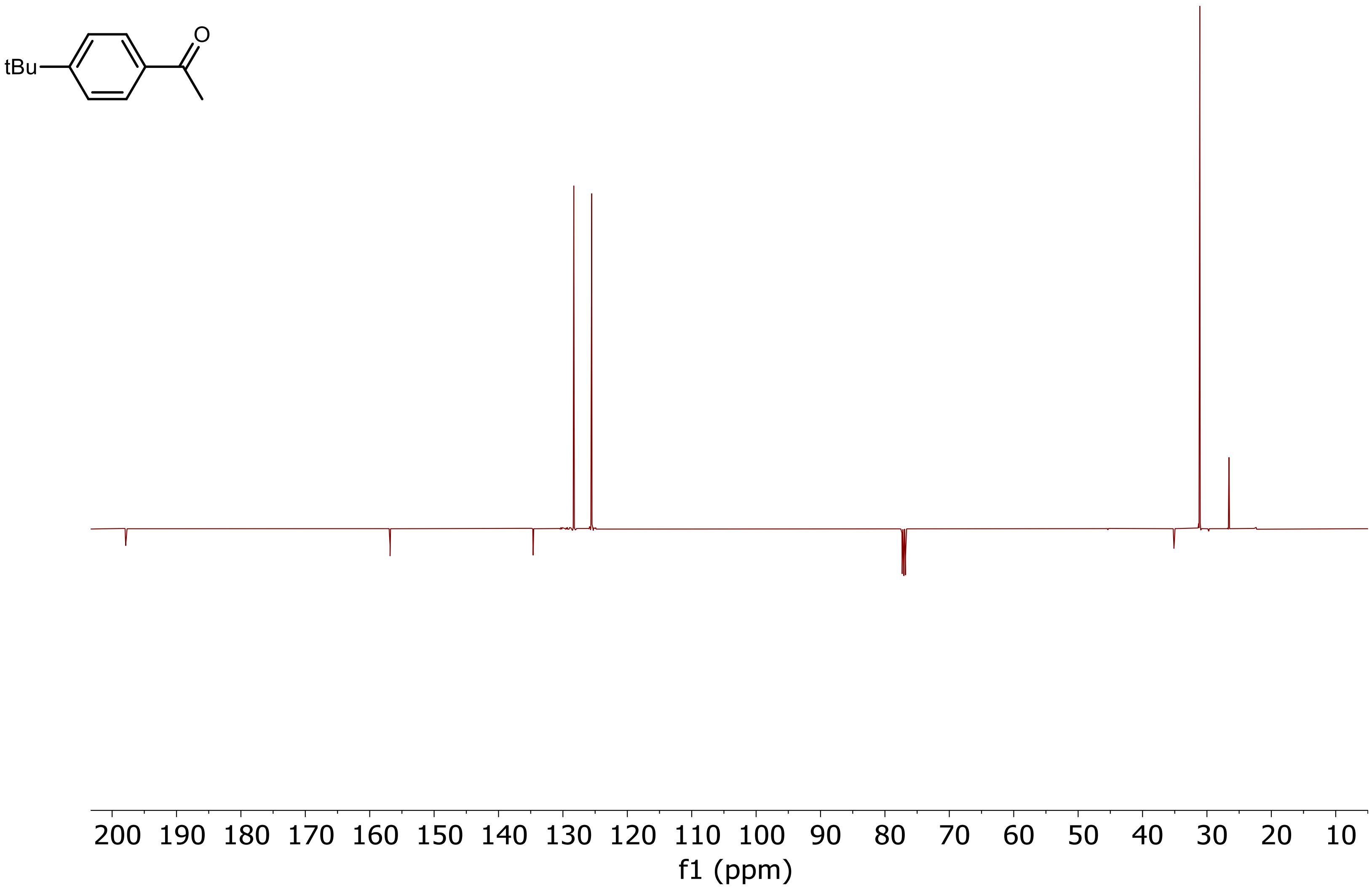

Figure S10. ^{13}C NMR spectrum of 4-*tert*buthylacetophenone in $CDCl_3$.

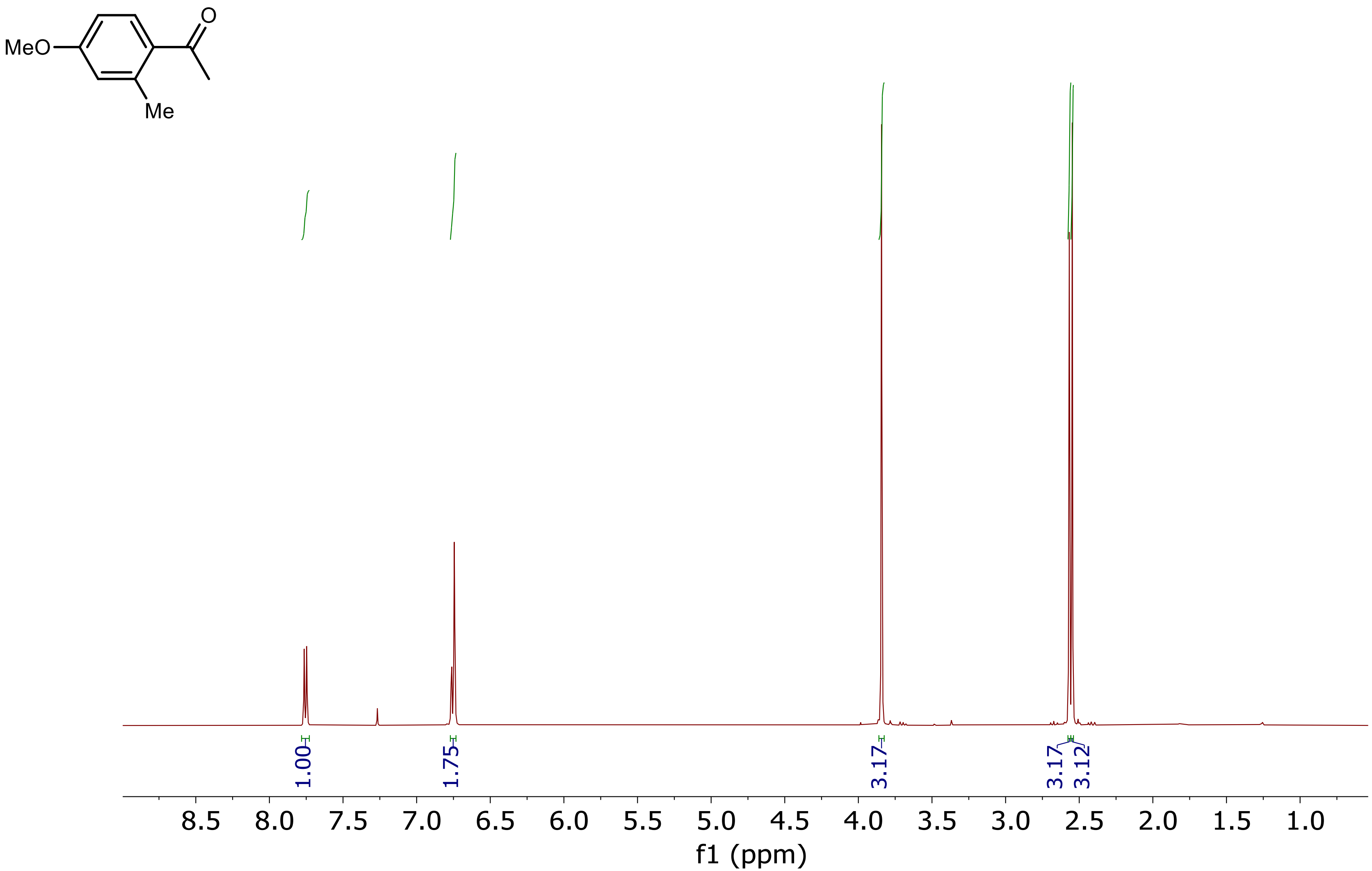

Figure S11. ^{1}H NMR spectrum of 4-methoxy-2-methylacetophenone in $CDCl_3$.

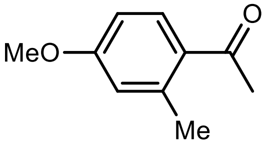

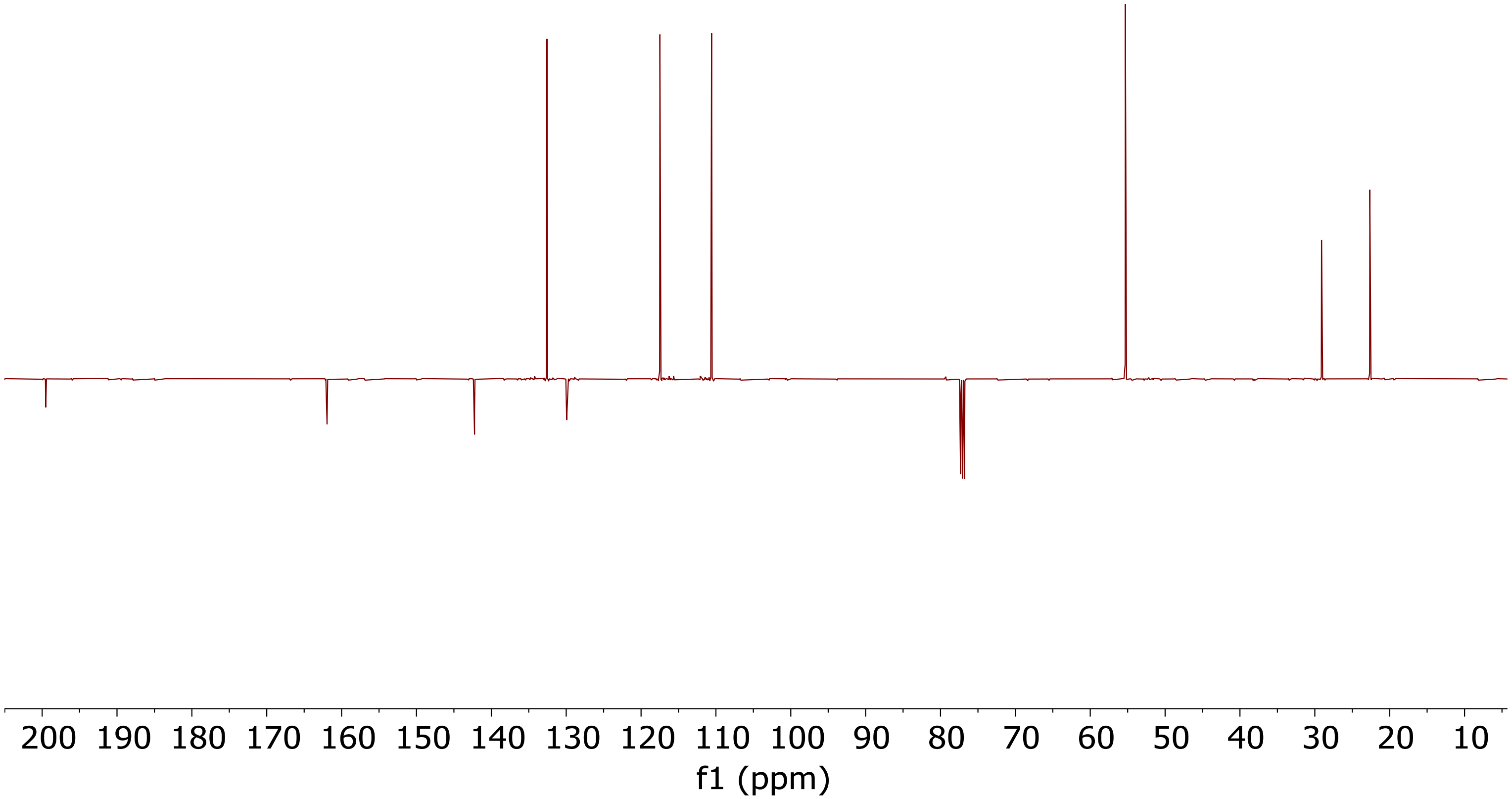

Figure S12. ^{13}C NMR spectrum of 4-methoxy-2-methylacetophenone in $CDCl_3$.

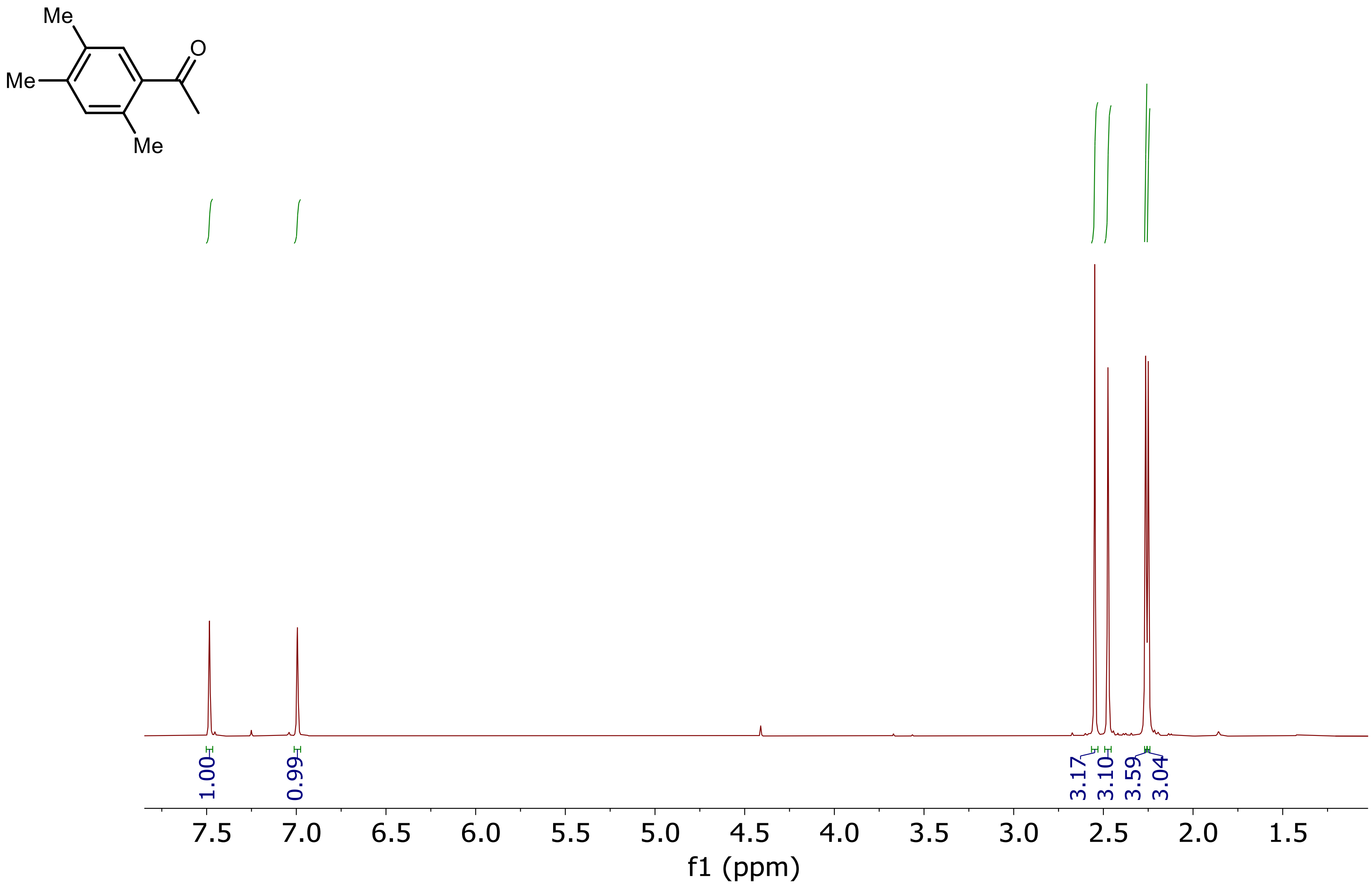

Figure S13. ^{1}H NMR spectrum of 2,4,5-trimethylacetophenone in $CDCl_3$.

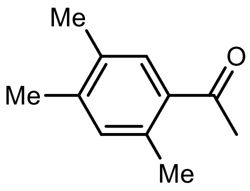

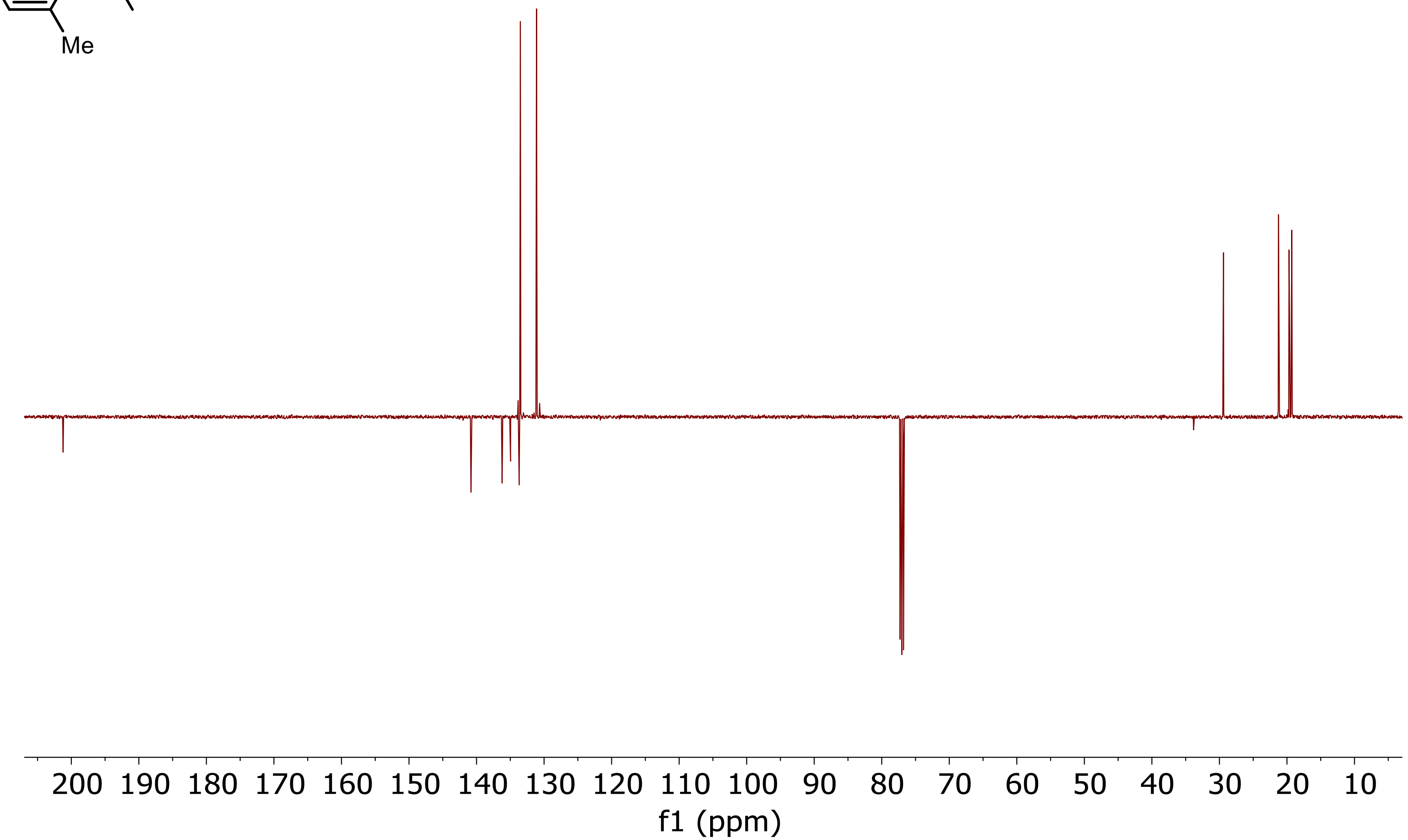

Figure S14. ^{13}C NMR spectrum of 2,4,5-trimethylacetophenone in $CDCl_3$.

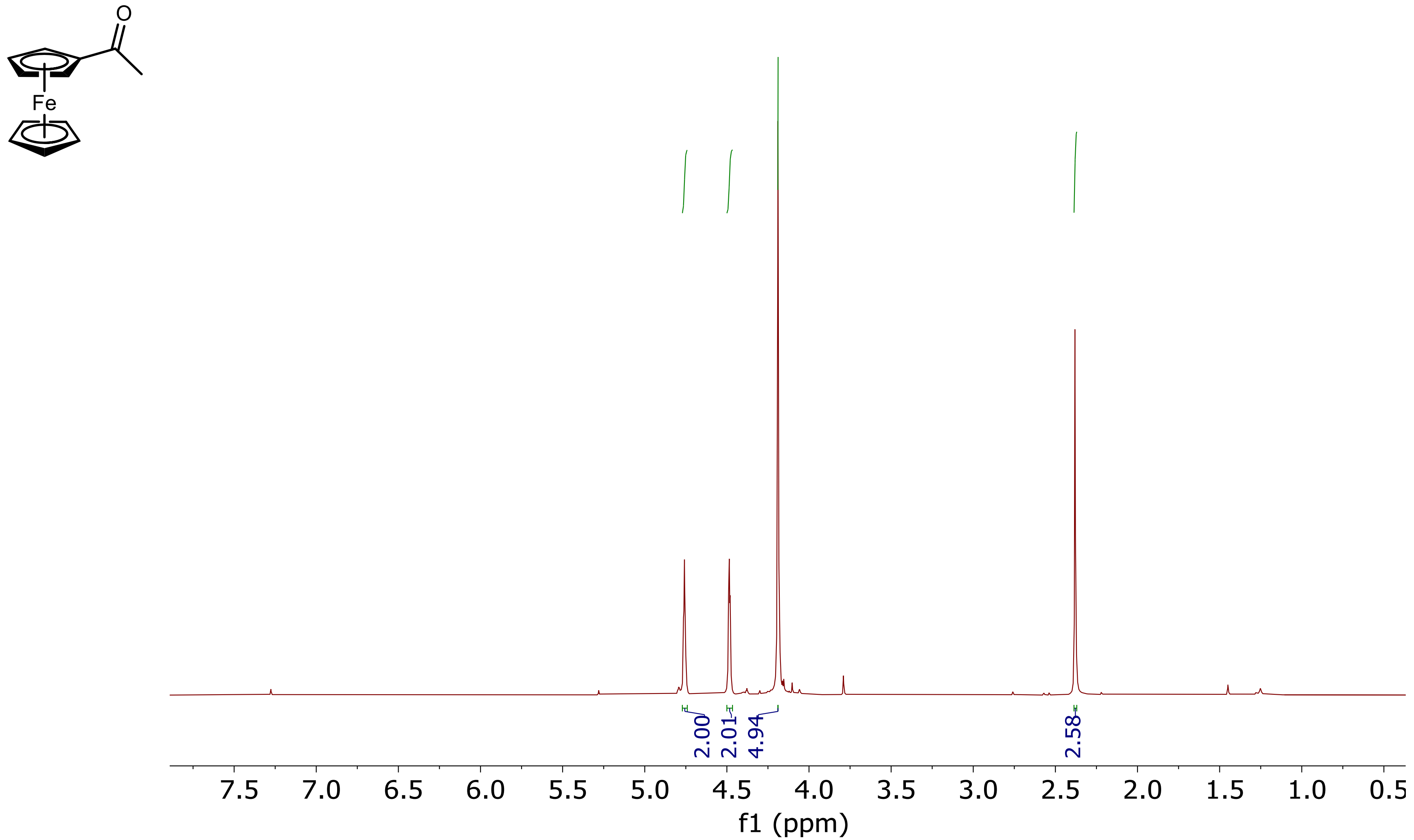

Figure S15. ^{1}H NMR spectrum of acetylferrocene in $CDCl_3$.

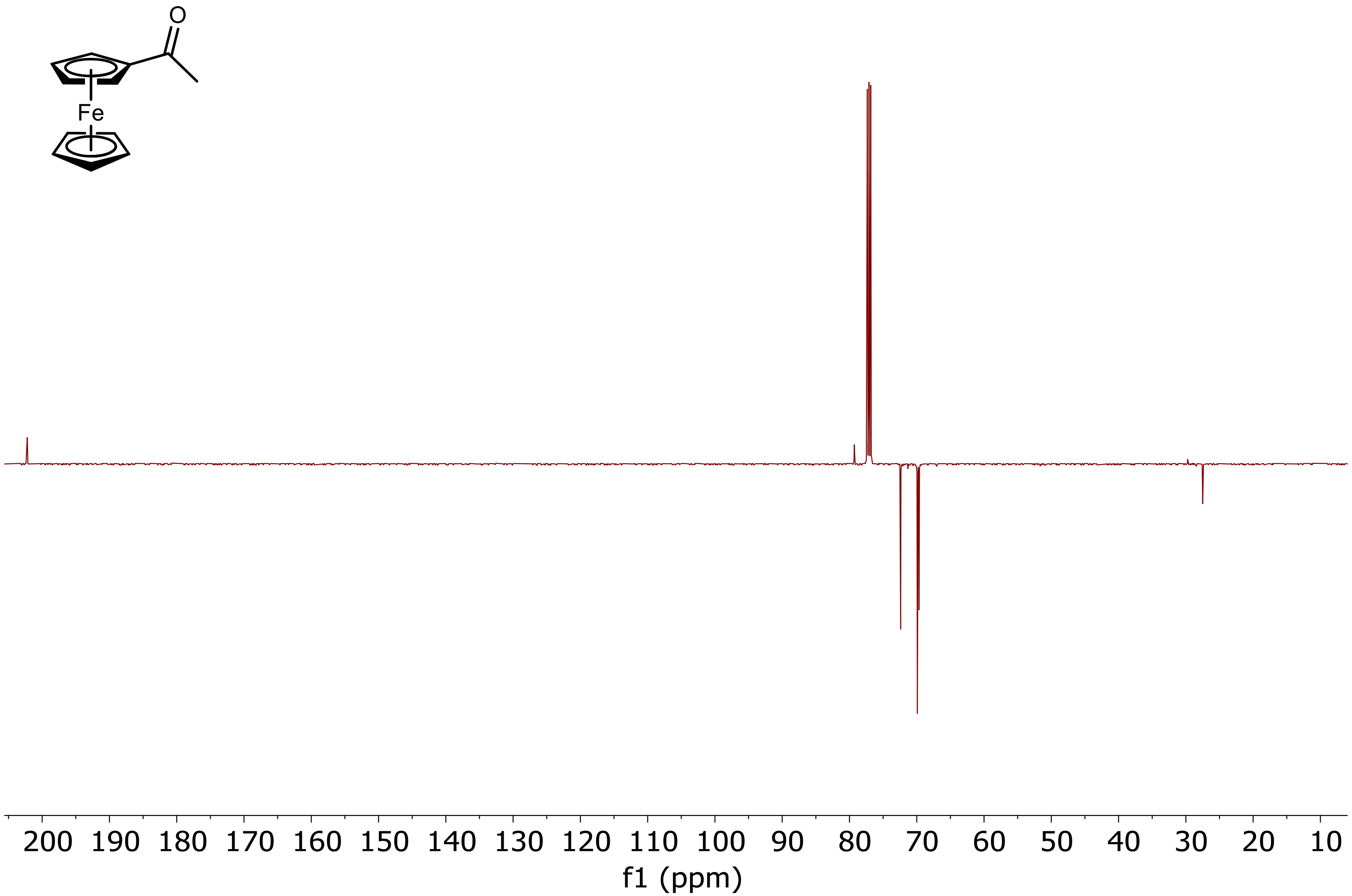

Figure S16. ^{13}C NMR spectrum of acetylferrocene in $CDCl_3$.

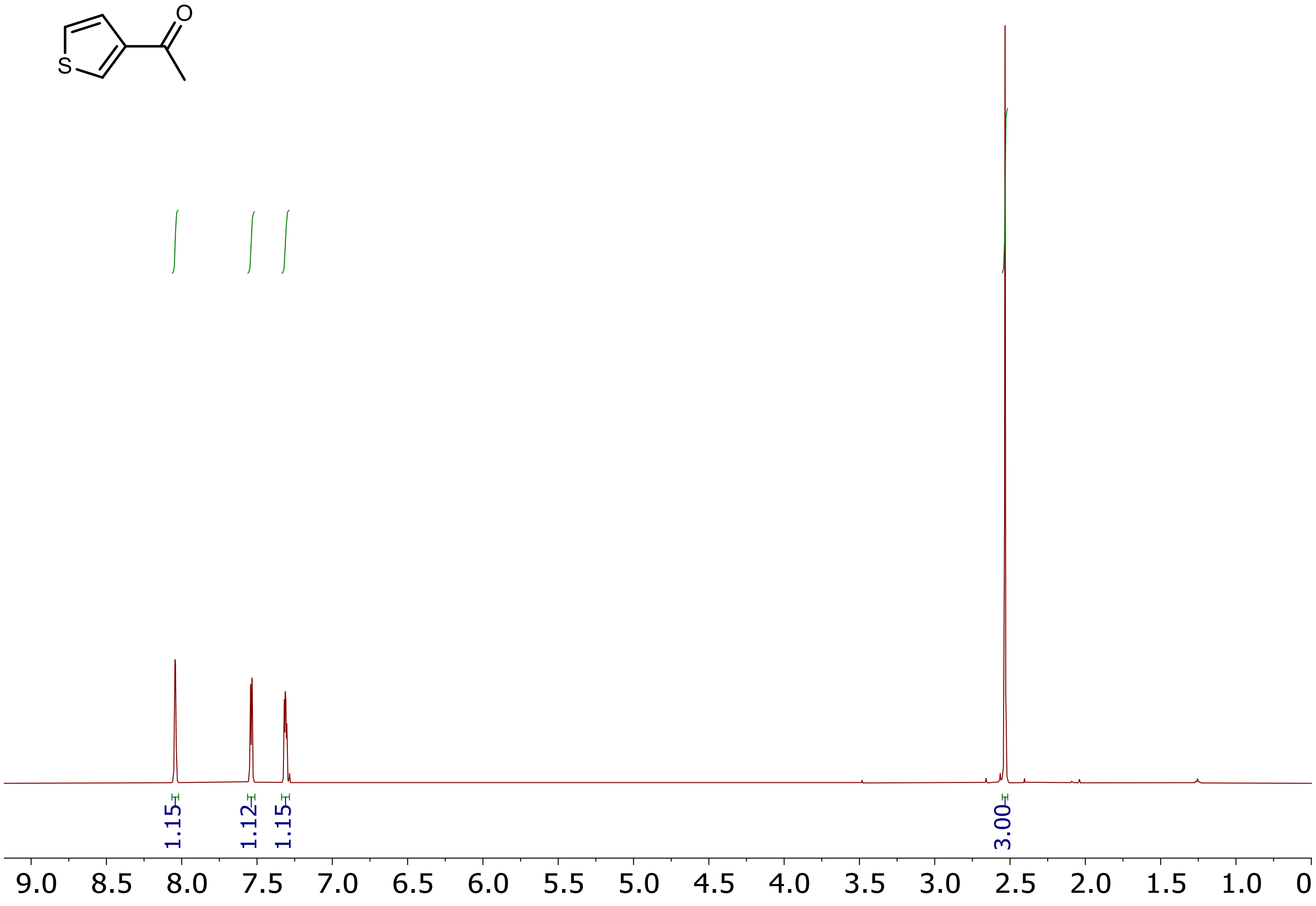

Figure S17. ^{1}H NMR spectrum of 3-acetylthiophene in $CDCl_3$.

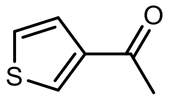

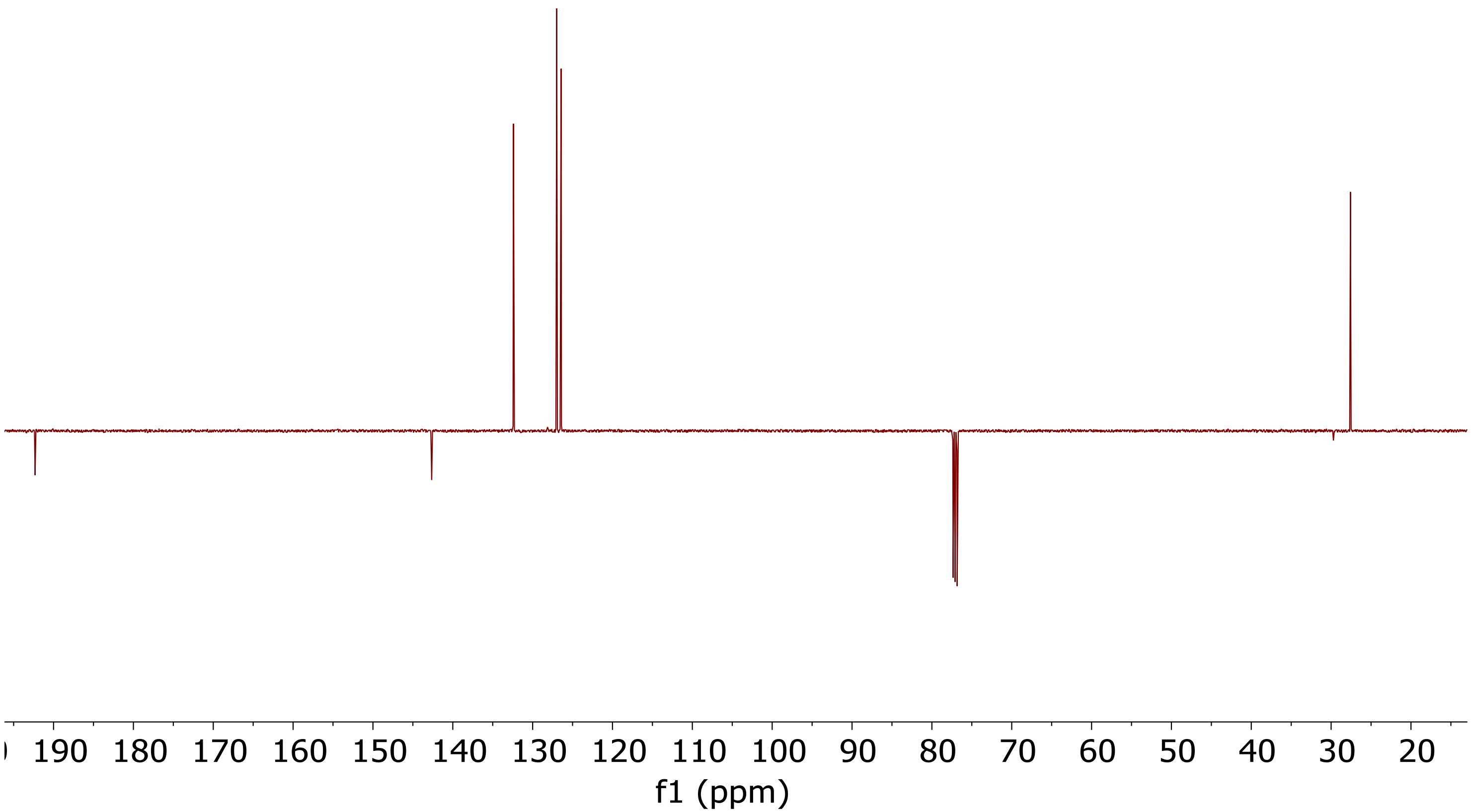

Figure S18. ^{13}C NMR spectrum of 3-acetylthiophene in $CDCl_3$.

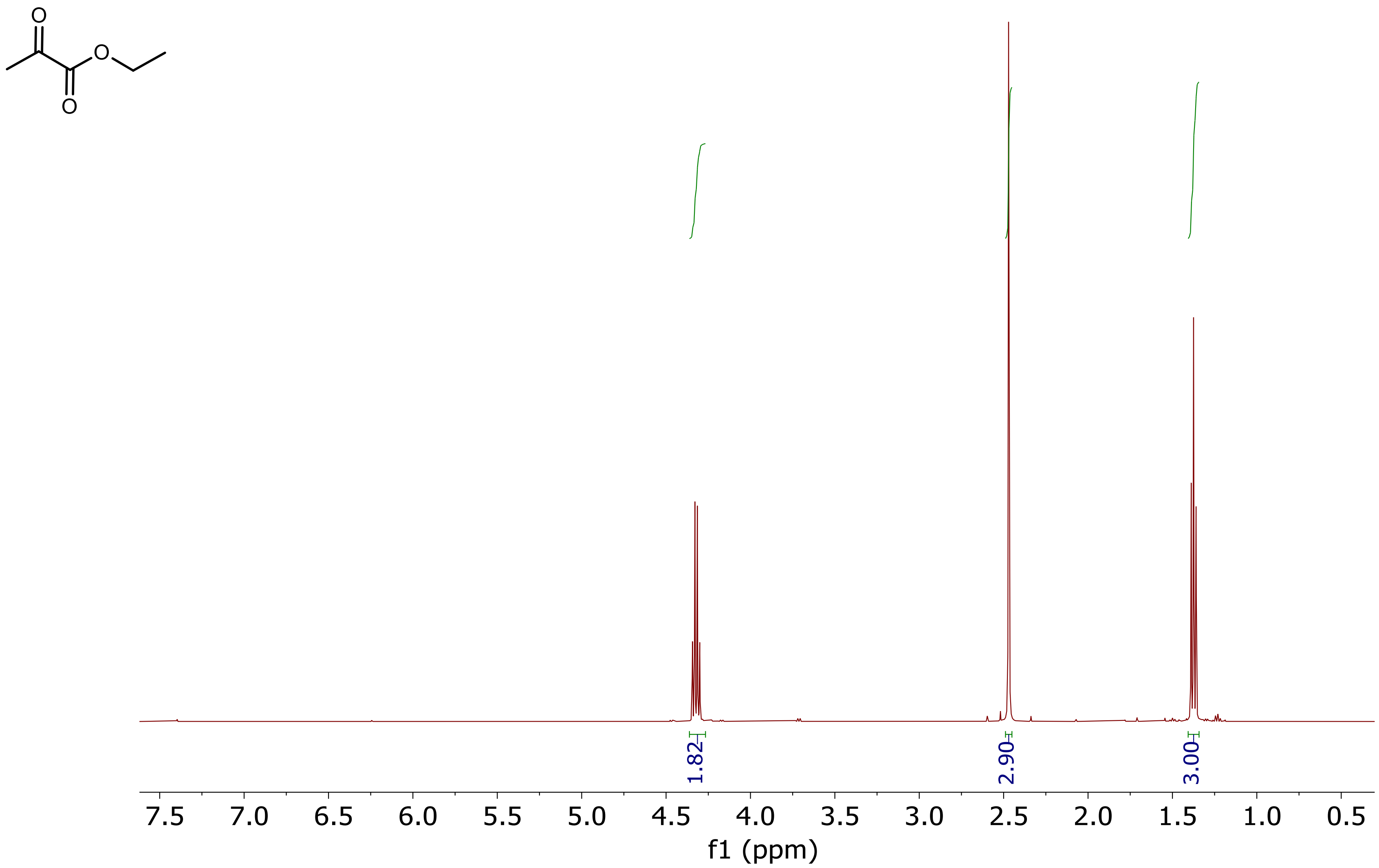

Figure S19. ^{1}H NMR spectrum of ethyl-pyruvate in $CDCl_3$.

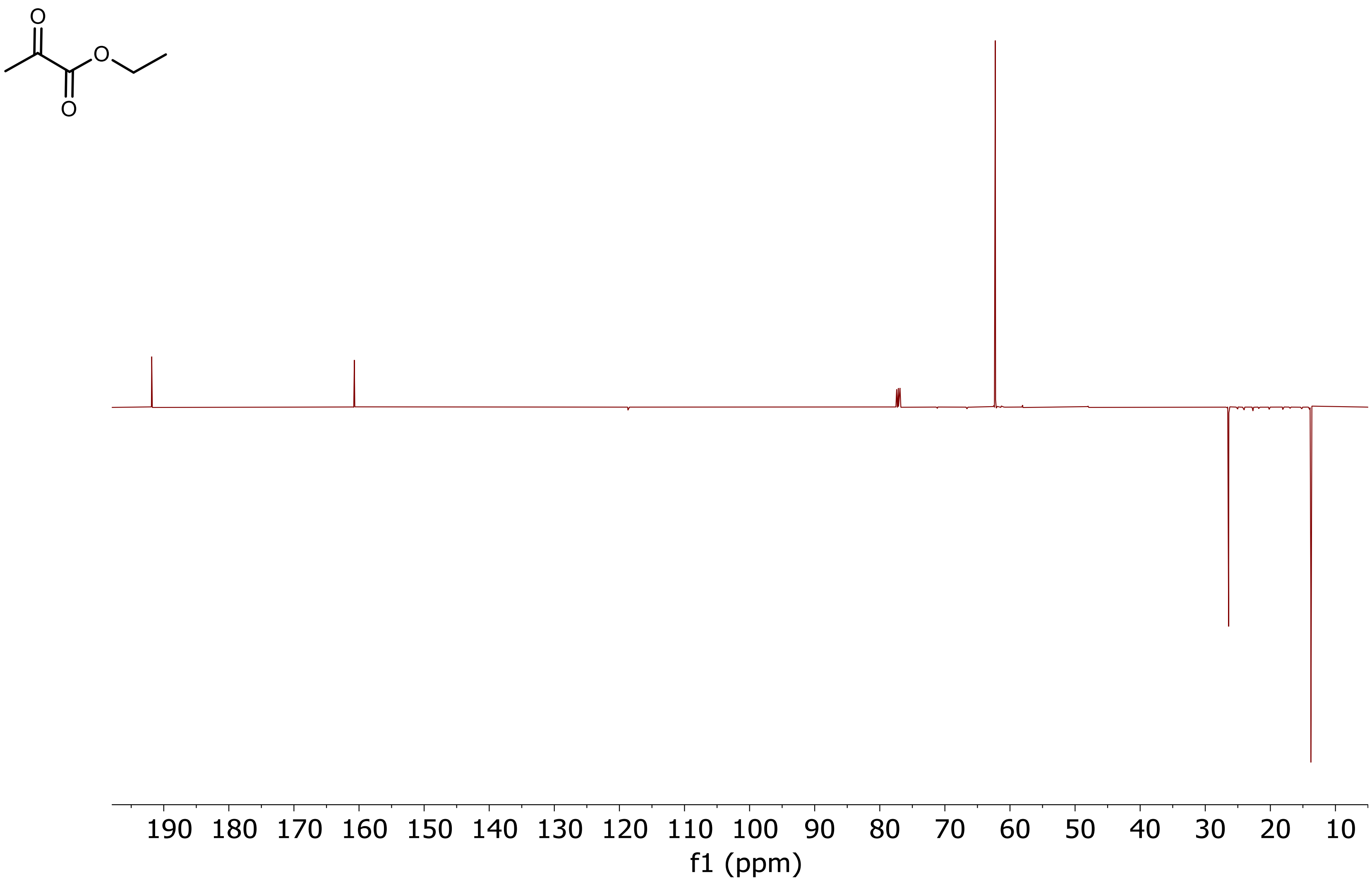

Figure S20. ^{13}C NMR spectrum of ethyl-pyruvate in $CDCl_3$.

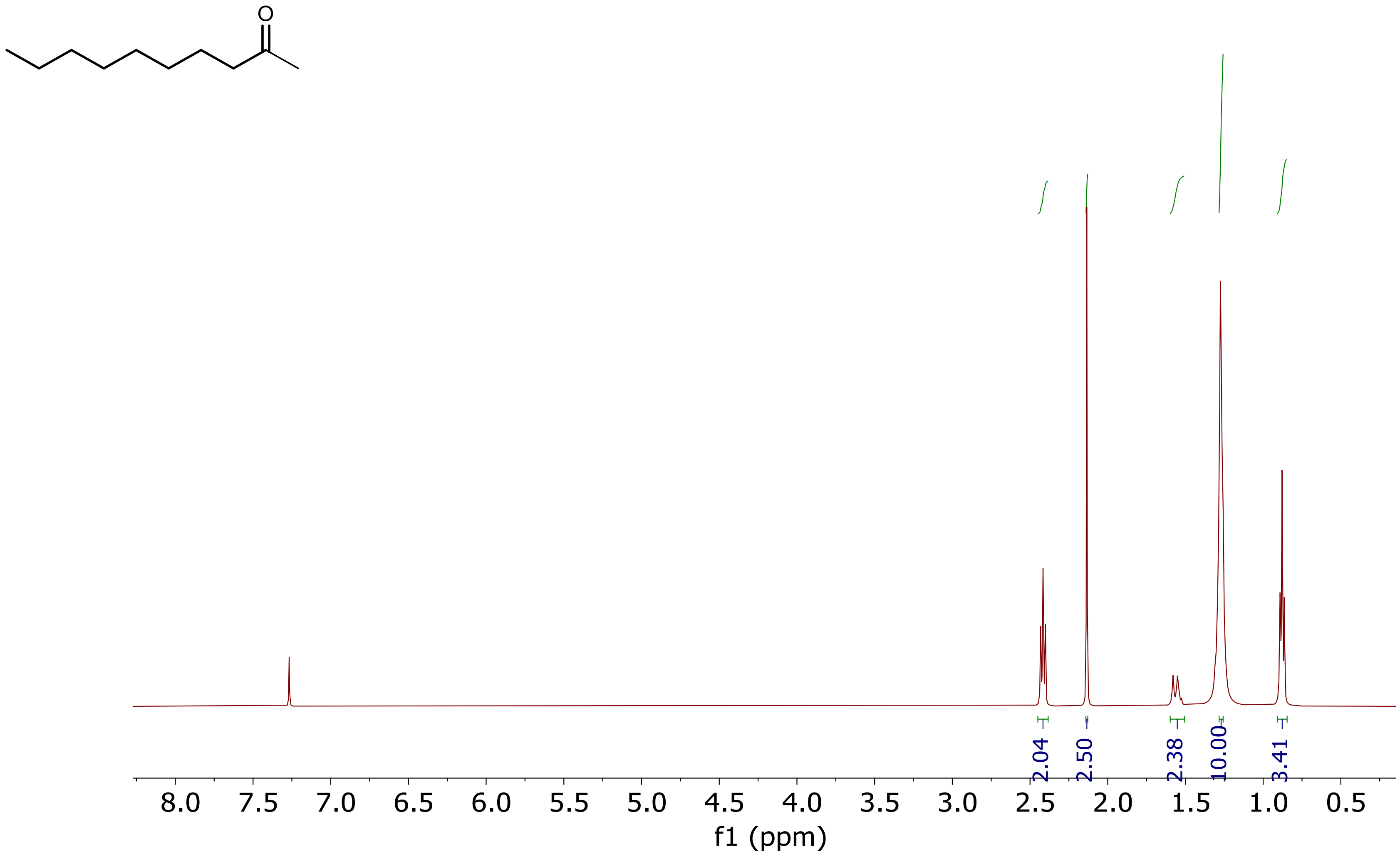

Figure S21. ^{1}H NMR spectrum of 2-decanone in $CDCl_3$.

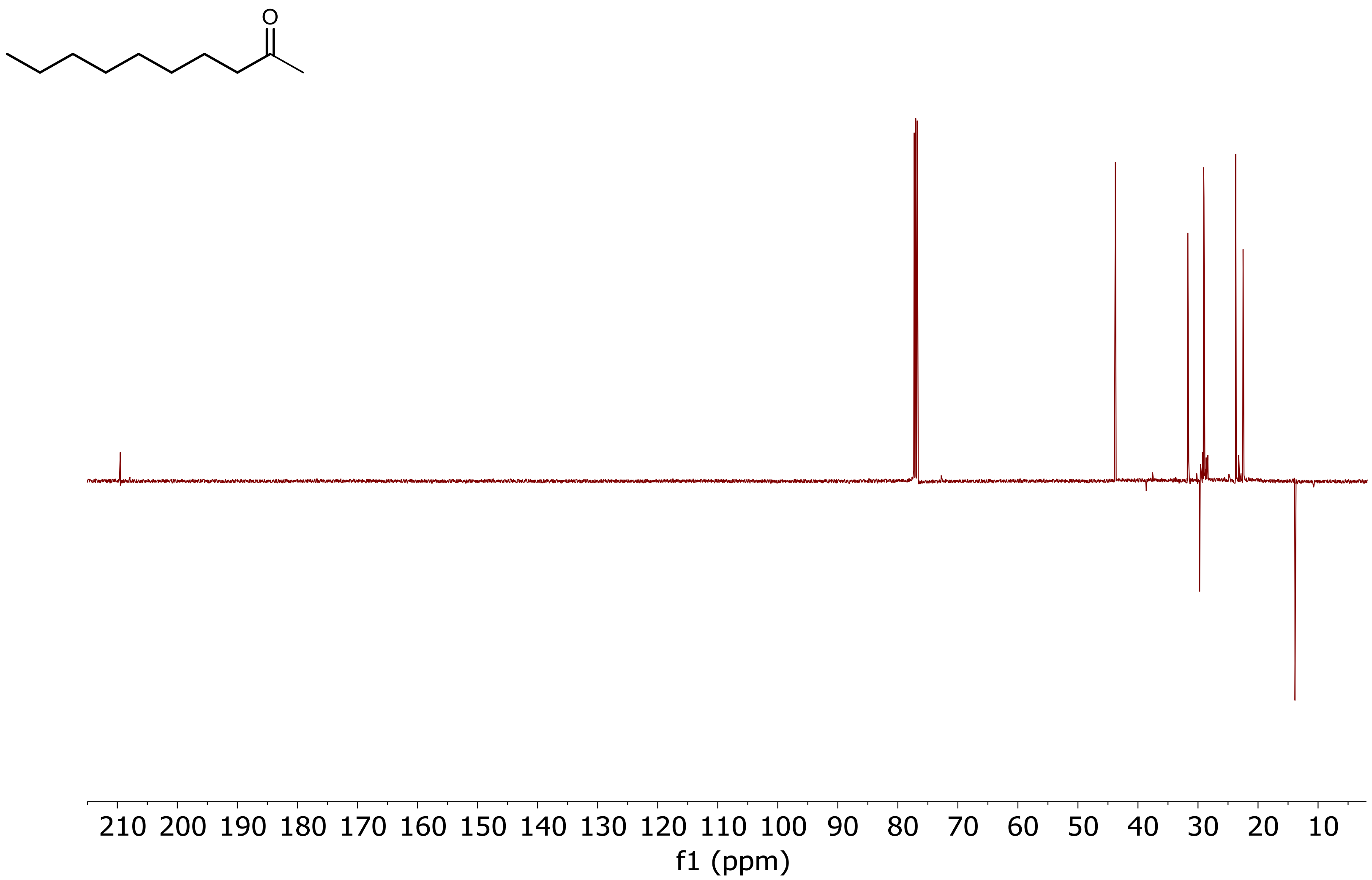

Figure S22. ^{13}C NMR spectrum of 2-decanone in $CDCl_3$.

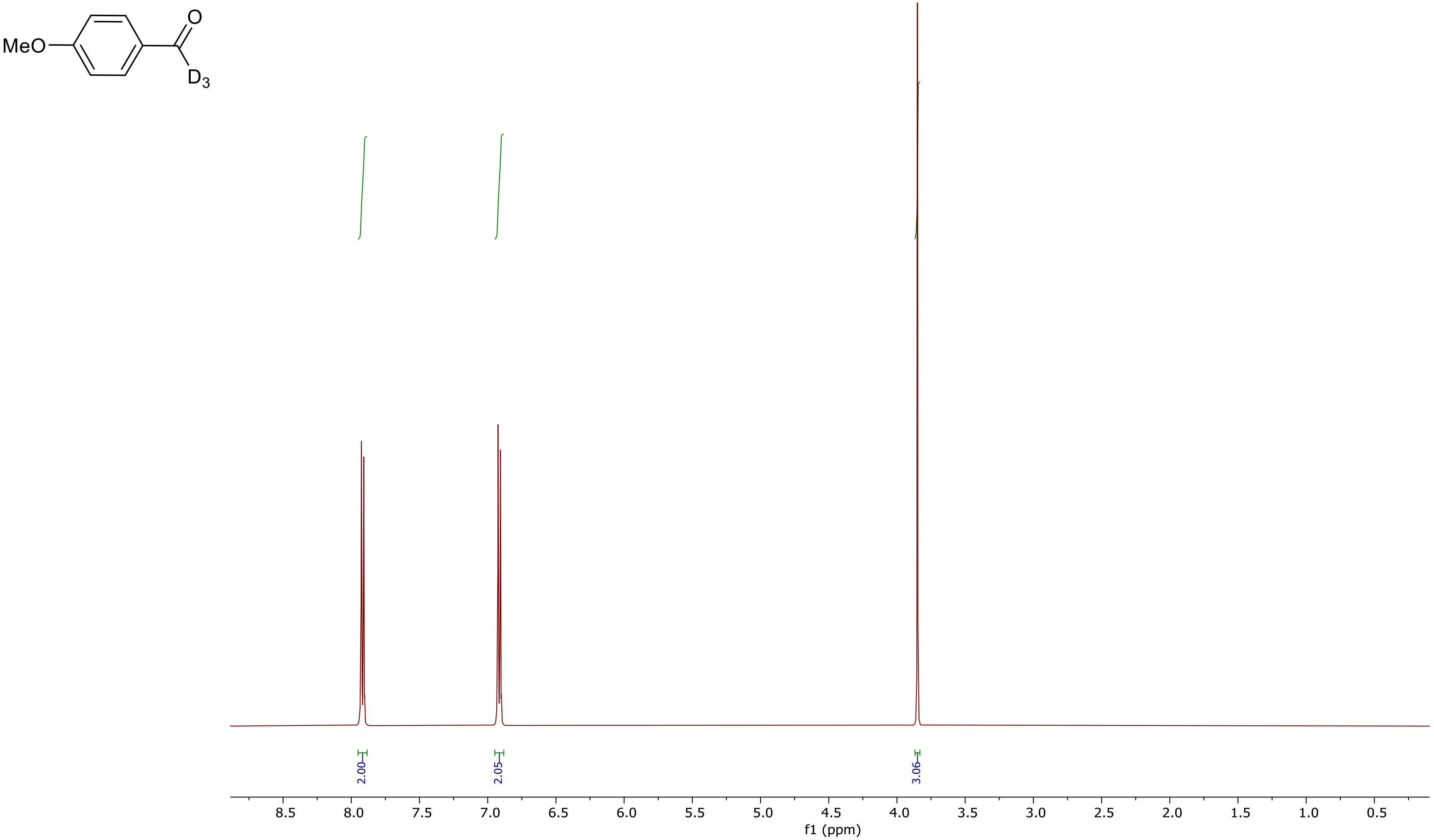

Figure S23. ^{1}H NMR spectrum of 1-(4-methoxyphenyl)ethan-1-one-2,2,2-*d3*

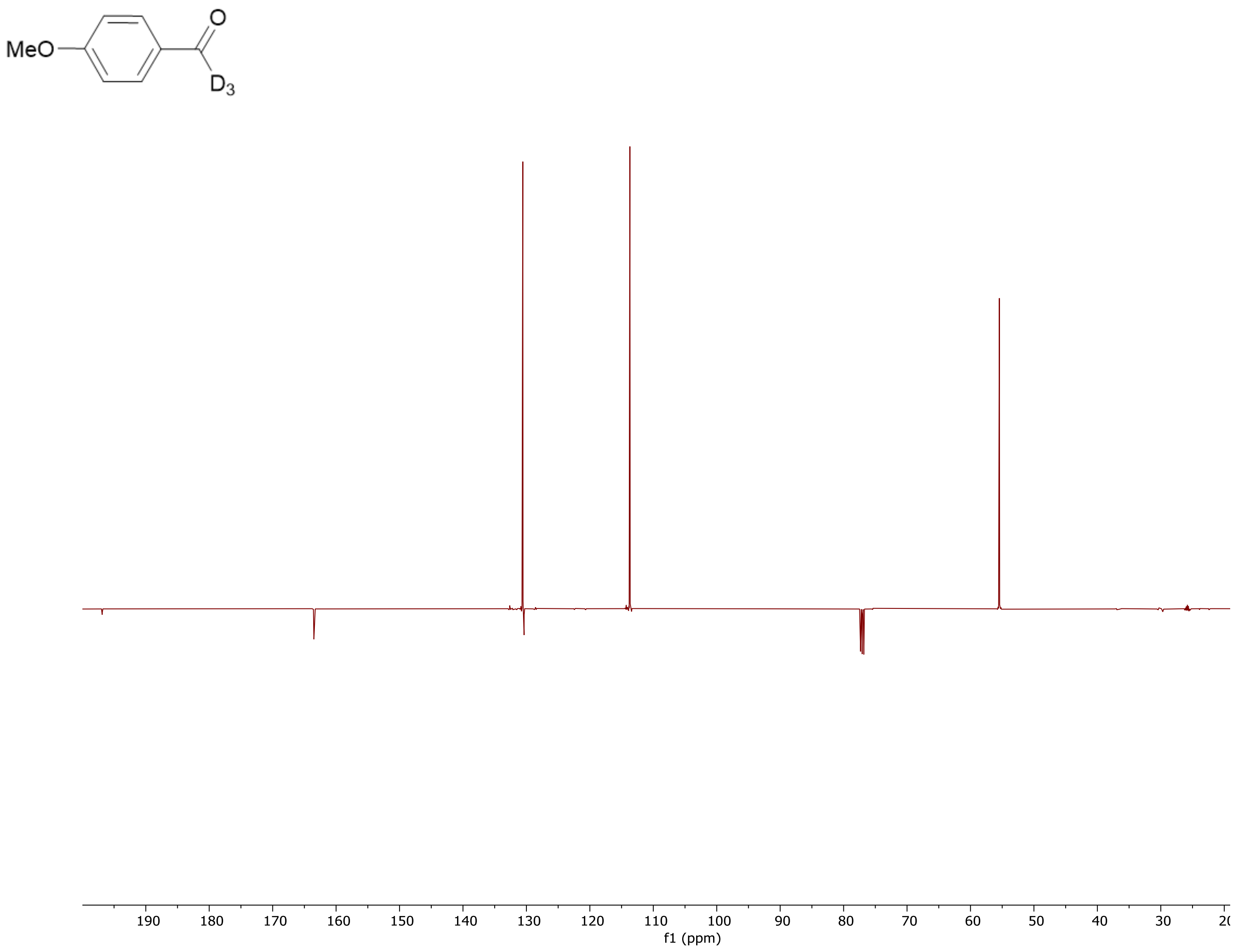

Figure S24. ^{13}C NMR spectrum of 1-(4-methoxyphenyl)ethan-1-one-2,2,2-*d3*

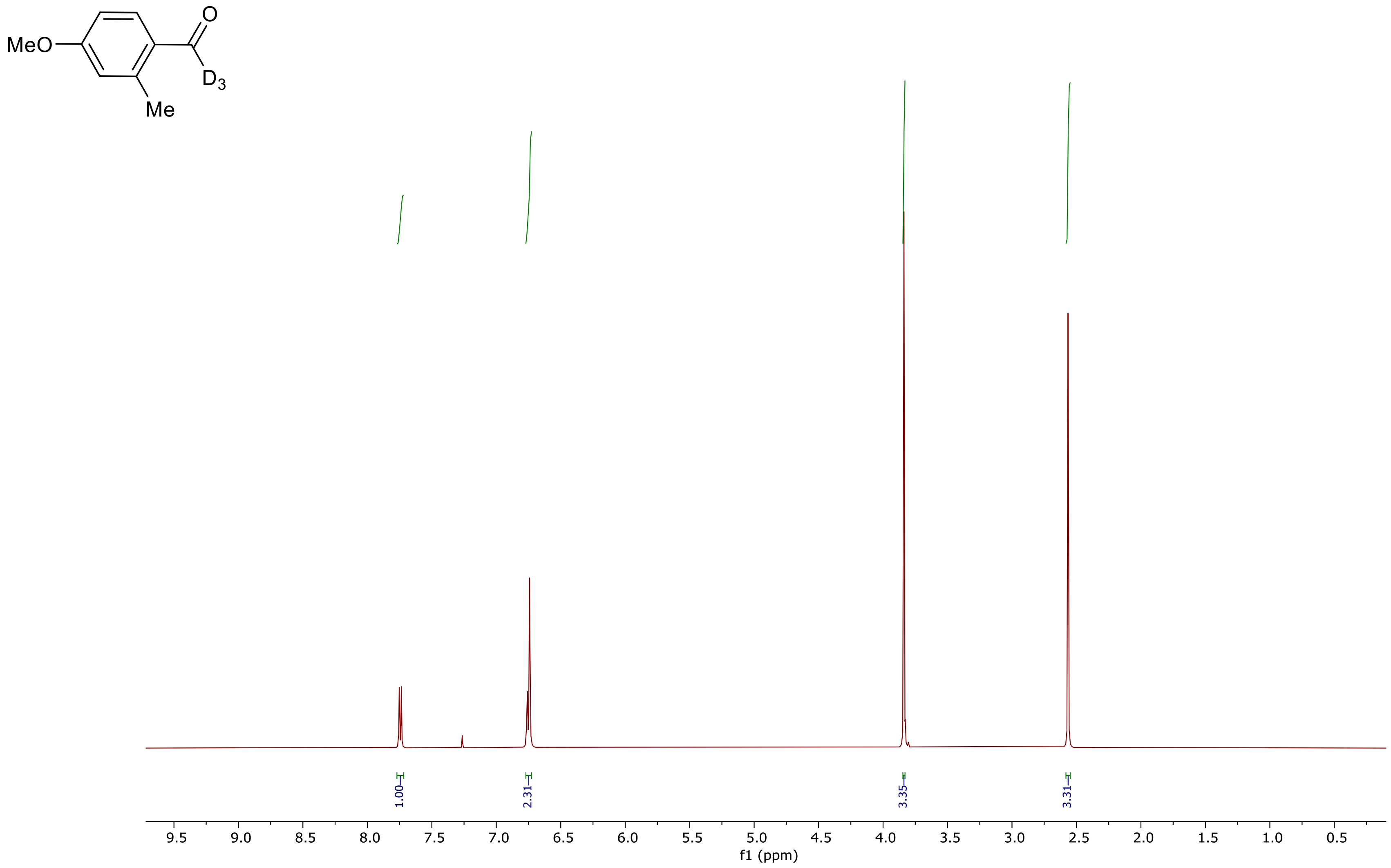

Figure S25. ^{1}H NMR spectrum of 1-(4-methoxy-2-methylphenyl)ethan-1-one-2,2,2-d3

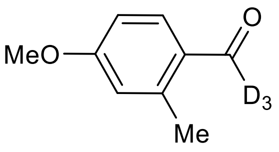

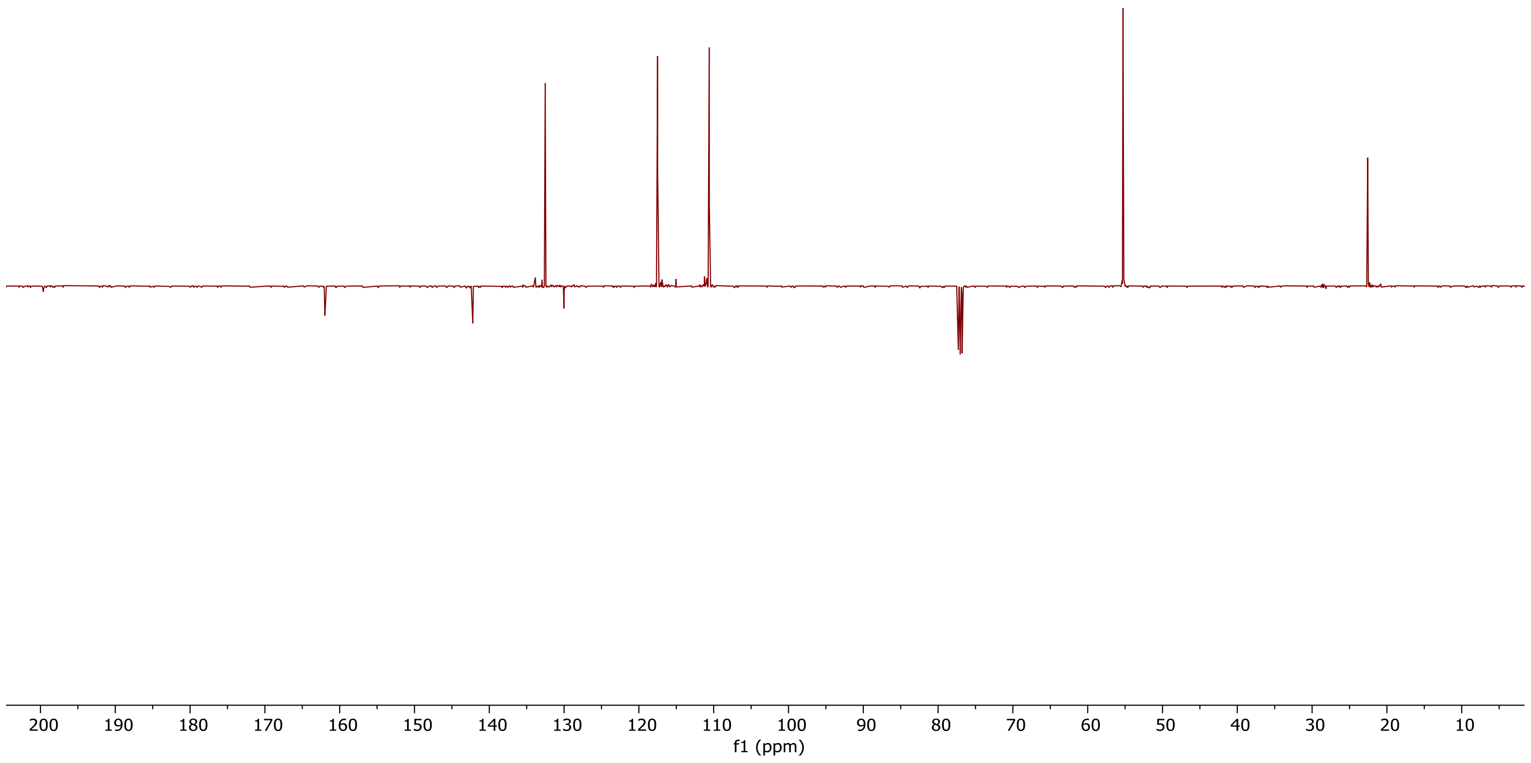

Figure S26. ^{13}C NMR spectrum of 1-(4-methoxy-2-methylphenyl)ethan-1-one-2,2,2-d3

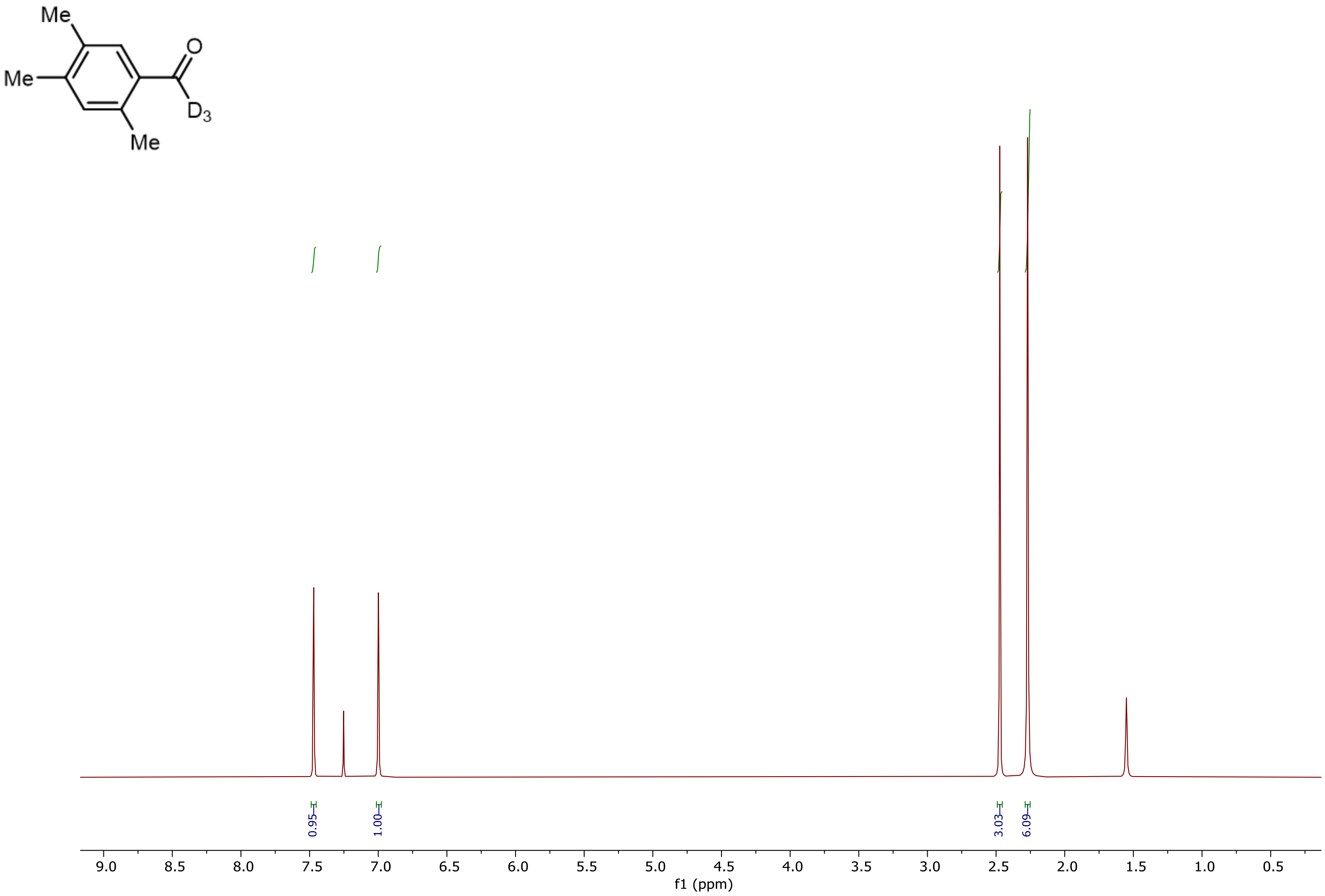

Figure S27. ^{1}H NMR spectrum of 1-(2,4,5-trimethylphenyl)ethan-1-one-2,2,2-d3

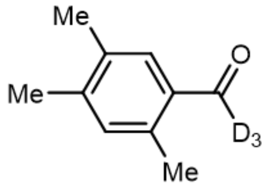

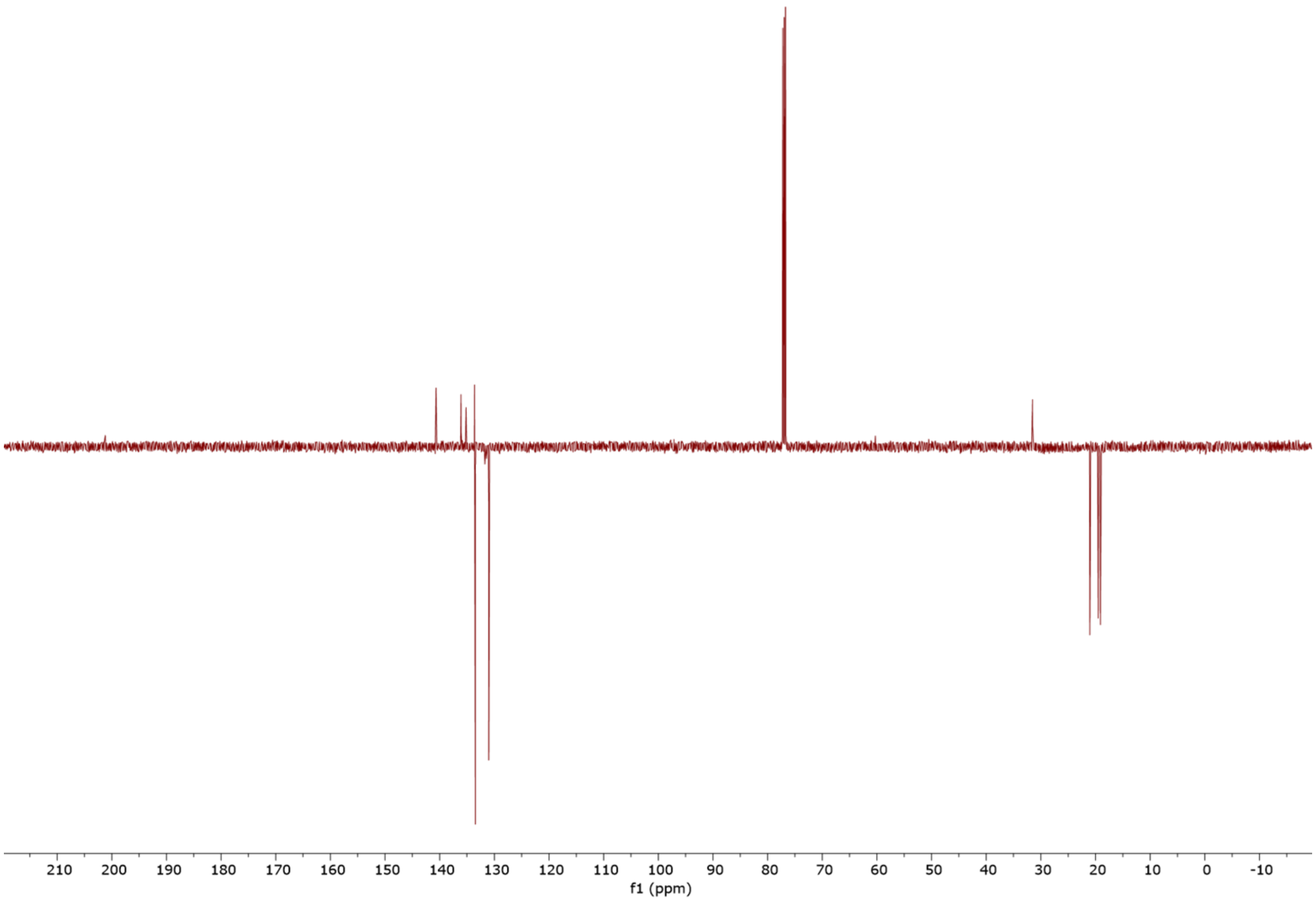

Figure S28. ^{13}C NMR spectrum of 1-(2,4,5-trimethylphenyl)ethan-1-one-2,2,2-d3

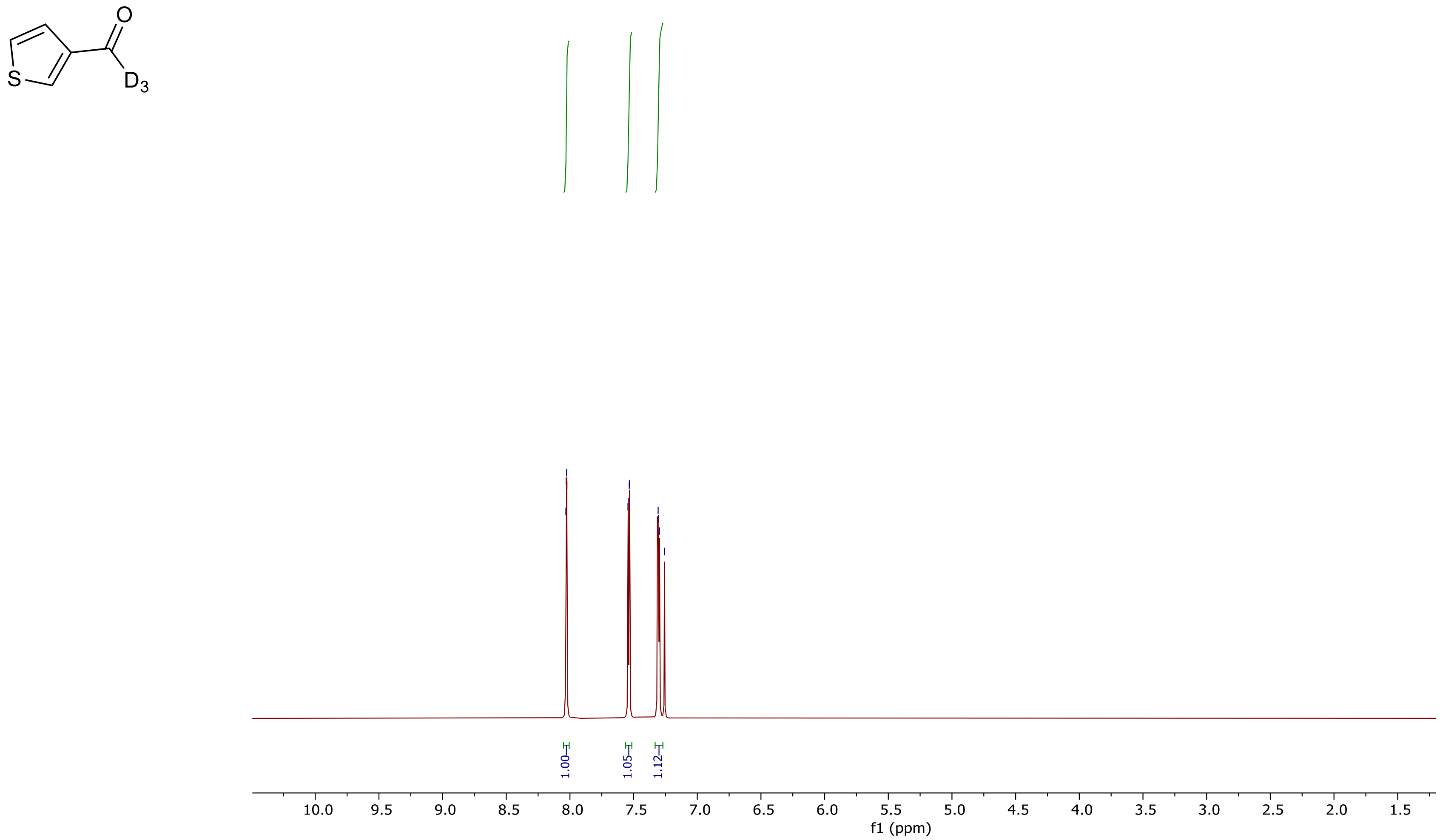

Figure S29. ^{1}H NMR spectrum of 1-(thiophen-3-yl)ethan-1-one-2,2,2-d3

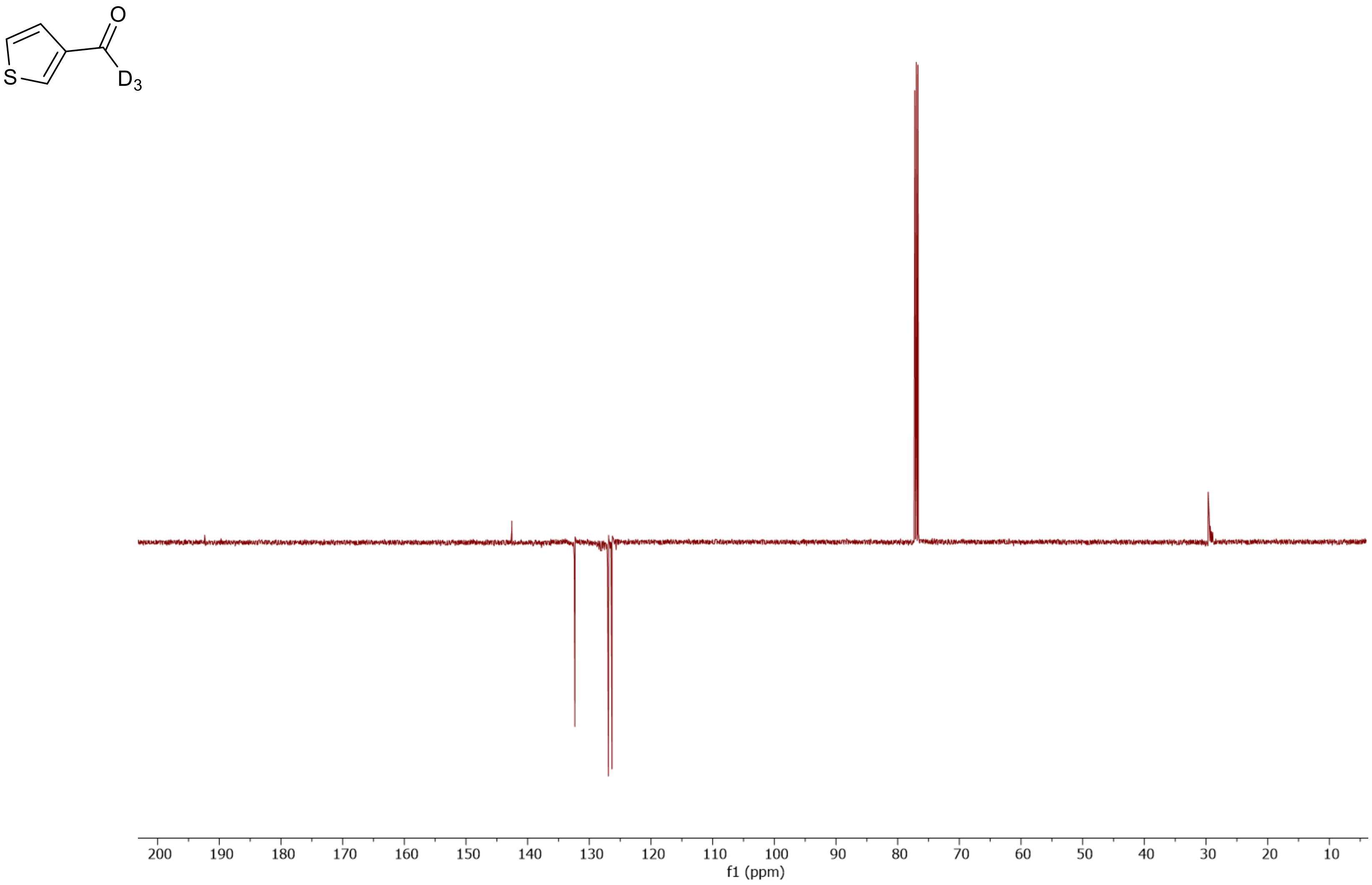

Figure S30. ^{13}C NMR spectrum of 1-(thiophen-3-yl)ethan-1-one-2,2,2-d3

3. References

1 D. T. Genna, G. H. Posner, *Org. Lett.*, **2011,** *13*, 5358-5361

2 G. Oss, J. Ho, T. V. Nguyen, *Eur. J. Org. Chem.*, **2018**, *29*, 3974-3981

3 F. Li, N. Wang, L. Lu, G. Zhu, *J. Org. Chem*. **2015**, *80*, 7, 3538–3546]

4 K. Fujita, R. Tamura, Y. Tanaka, M. Yoshida, M. Onoda, R. Yamaguchi *ACS Catal.*, **2017**, *7*, 7226–7230

5 G. L. Sorella, L. Sperni, P. Ballester, G. Strukul, A. Scarso, *Catal. Sci. Technol.*, **2016**, *6*, 6031-6036

6 R. Das, D. Chakraborty, *Appl. Organometal. Chem.*, **2012**, *26*, 722-726

7 W. Liu, H. Wang, CJ. Li, *Organic Letters*, **2016,** *18*, 2184-2187

8 M. Hassam, WS. Li, *Tetrahedron*, **2015**, *71*, 2719-2723

9 M. Głodek, A. Makal, P. Paluch, M. Kadziołka-Gaweł, Y. Kobayashi, J. Zakrzewski, D. Plażuk, *Dalton Trans.***,** **2018**, **47**, 6702-6712

10 A. Lopalco, G. Dalwadi, S. Niu, R. L. Schowen, Justin Douglas,V. J. Stella, *J. Pharm. Sci.*, **2016**, *105*, 705-713

11 Y. M. A. Yamada, C. K. Jin, Y. Uozumi, *Org. Lett.*, **2010**, *12*, 4540–4543

12. R. Giles, G. Ahn, K. W. Jung *Tetrahedron Letters*, **2015**, *56*, 6231–6235
